# Molecular Identification of Human and Plant Pathogens in Municipal Domestic Wastewater for Hydroponic System Applications

**DOI:** 10.1155/ijm/6958575

**Published:** 2025-11-25

**Authors:** Lukas Simon Kriem, Neil King, Sinja Niemann, Yevhen Vainshtein, Mirko Sonntag

**Affiliations:** ^1^Department for Water Technologies, Resource Recovery and Scale-Up, Fraunhofer-Institut für Grenzflächen- und Bioverfahrenstechnik IGB, Stuttgart, Baden-Württemberg, Germany; ^2^Department for In-Vitro Diagnostics, Fraunhofer-Institut für Grenzflächen- und Bioverfahrenstechnik IGB, Stuttgart, Baden-Württemberg, Germany

**Keywords:** hydroponic systems, metagenomic analysis, PCR detection, wastewater pathogens, water reuse

## Abstract

Water is essential for human survival and socioeconomic development, yet its overconsumption threatens global food security and ecosystem integrity. This necessitates a 60% increase in food production, further straining water resources. Hydroponic systems represent a promising solution, utilizing up to 90% less water than traditional methods while providing optimal growing conditions for crops. This study was aimed at developing a PCR-based detection system for main human and plant pathogens in hydroponic systems using treated domestic wastewater. Metagenomic analysis of wastewater samples revealed significant microbial diversity, identifying human pathogens such as *Pseudomonas aeruginosa* and *Yersinia enterocolitica*, alongside plant pathogens including *Rhodococcus fascians*. Specific primer pairs for the most abundant species found in a domestic municipal wastewater sample of target pathogens (*Streptococcus mutans*, *P. aeruginosa*, *Acinetobacter baumannii*, *Y. enterocolitica*, *Enterococcus faecalis*, *Pseudomonas viridiflava*, *R. fascians*, *Xanthomonas vesicatoria*, and *Pseudomonas syringae*) were designed and validated, ensuring high specificity and efficiency. Future research should focus on enhancing detection methods and optimizing DNA extraction techniques to improve pathogen quantification and management in hydroponic systems. This approach is crucial for sustainable agricultural practices that minimize water usage while ensuring food safety and environmental health.

## 1. Introduction

Over the next few decades, humanity faces significant challenges in achieving sustainable food production, responsible management of water and resources, and establishing closed material cycles. Hydroponic systems could play an important role in tackling the global problem of scarce water resources in food production by enabling the cultivation of plants using up to 90% less water [1]. Hydroponic systems have gained considerable attention for their capacity to facilitate plant growth in controlled settings. This soil-less approach to cultivation relies on water-based nutrient solutions to deliver essential elements for healthy plant development directly to the plant roots. Hydroponics provides numerous benefits compared to conventional soil-based farming, including efficient use of resources, optimized nutrient uptake, and higher crop yields [2, 3].

In addition to their use in traditional agriculture, hydroponic systems offer significant potential for wastewater treatment and wastewater reuse. Unlike conventional hydroponics, wastewater hydroponics recover nutrients from wastewater instead of using a standard nutrient solution, enabling the purification of wastewater [4]. They are often referred to as bioponics. Most wastewater contains organic contaminants such as nitrogen, phosphorus, calcium, magnesium, and potassium. These nutrients serve as essential elements for plant growth.

Crops cultivated in these hydroponic systems are seen to have fewer food safety concerns because they have been shown to reduce common microbial contaminants found in soil, wild and farm animals, and pests. Further, they typically show reduced chemical contaminants such as pesticides and soil fertilizers [5]. However, the use of wastewater for hydroponic systems, especially, poses a risk for contamination and food safety [6]. Pathogens of concern encompass enteric bacteria, viruses, protozoa, parasitic worms, and their eggs. Originating from human communities, domestic wastewater comprises potential pollutants like detergents and oils, along with human wastes such as feces and urine. Feces are the main source of most human pathogens in wastewater. Additionally, industrial waste, notably from food production and animal processing, can also introduce pathogenic microorganisms into wastewater [7, 8].

Bacteria represent the most diverse category of human pathogens found in wastewater. Numerous bacterial strains inhabit the human intestine and are excreted in feces. While many of these bacteria are harmless or even beneficial to their hosts, some are pathogenic. Among them, enteric bacterial pathogens, such as *Salmonella* spp., *Escherichia* spp., *Shigella* spp., *Yersinia* spp., and *Vibrio cholerae*, make up the majority of bacterial pathogens present in wastewater. They commonly induce gastrointestinal infections, resulting in symptoms like diarrhea, dysentery, and gastroenteritis. Additional diseases caused by bacteria in wastewater encompass wound infections (*Pseudomonas aeruginosa*) and respiratory infections (*Legionella pneumophila* and *Mycobacterium avium*). Certain bacteria, like *L. pneumophila*, *M. avium*, *P. aeruginosa*, and *Aeromonas hydrophila*, are environmental and function as opportunistic pathogens, causing disease in individuals compromised by factors such as a weakened immune system or reduced physical barriers due to wounds [8, 9].

Furthermore, wastewater can contain pathogenic species that threaten agricultural productivity in hydroponic systems, potentially limiting crop cultivation when wastewater is used for irrigation [10, 11]. Plant pathogens are microorganisms that infect plants and cause diseases, having evolved specifically to invade and multiply within host plants [12, 13]. They can infiltrate plants through wounds or natural openings [12]. The presence of plant pathogens in irrigation water has been recognized as a significant issue, which has intensified as agriculture increasingly relies on recycled water [14]. Hydroponic systems provide some protection against diseases typically transmitted through soil or animals in open fields. However, these systems do not fully guarantee plant health. In cases of contamination, plant pathogens can rapidly spread through the circulating water, infecting all plants in the system simultaneously [15].

In order to clarify the water reuse potential, the European Union has recently taken proactive steps to address this challenge by introducing Regulation 2020/741 that looks at minimum requirements for water reuse [16]. This regulation establishes a standardized framework within the European Union for managing the risks associated with agricultural water reuse, including the monitoring of certain microorganisms. While the regulations focus on the log removal of certain microorganisms (log 6 removal for viruses, log 5 removal for bacteria such as *Escherichia coli*, and log 4 removal for protozoa such as *Clostridium perfringens*), it lacks regulation for other human and plant pathogens that are found in wastewater. Nevertheless, tracking some major human pathogens will help in the risk assessment of hydroponic units for human health on the consumption of potentially contaminated crops while tracking plant pathogens helps to control potential disease outbreaks within a cultivation system.

Thus, tracking human and plant pathogens in wastewater intended for agricultural reuse is essential to prevent health risks, ensure environmental safety, and maintain agricultural productivity. Regular monitoring, advanced treatment methods, and thorough risk assessments are critical components of a safe and sustainable wastewater reuse strategy. Human pathogens such as the genera *Roseomonas*, *Methylobacterium*, and *Bacillus* [17], along with plant pathogens, can persist in recycled water and threaten both crop health and consumer safety. Monitoring these microorganisms is challenging due to their low concentrations, complex water matrices, and the presence of PCR inhibitors like humic acids and detergents [18]. Traditional detection methods often lack the sensitivity or specificity needed for reliable surveillance in such environments.

Existing methods for detecting microorganisms in liquid samples include culture-based techniques, microscopy, and molecular methods such as quantitative PCR (qPCR) and next-generation sequencing (NGS) [19]. Culture-based techniques, while traditional and inexpensive, often fail to detect viable but nonculturable (VBNC) organisms and are limited to fast-growing species and may not allow differentiation of species based on morphologies. Microscopy provides direct visualization but lacks species-level resolution and sensitivity. NGS offers high-throughput, detailed profiling of microbial communities but is costly, time-consuming, and impractical for routine monitoring. These limitations often result in inadequate sensitivity, particularly when target microorganisms are present in low concentrations or when environmental inhibitors interfere with DNA extraction and amplification.

At the same time, PCR-based detection systems allow frequent and tailored pathogen monitoring in treated wastewater used in hydroponic systems [20]. Unlike broad-spectrum NGS, this method utilizes specifically designed primers to detect select microorganisms. Compared to broader metagenomic approaches, PCR is more cost-effective, faster, and better suited for routine monitoring and practical field diagnostics, though it typically requires prior knowledge of the target organisms. Within the described molecular methods, subcategories further allow quantification (RT-qPCR) or detection of low quantities (droplet digital PCR [dPCR]). The development of such methods would allow better analysis of hydroponic systems, early detection of potential health risks, and evaluation of disinfection technologies.

All described PCR-based methods have in common that DNA-based primers need to be available to allow specific detection of microorganisms. While many species-specific primers have been previously described in publications, they are only fit for a specific research question and may not be appropriate for other applications. One downfall may be the detection of false positive results due to cross-reaction of primers with other microorganisms in a sample [21]. Further, the described primers have been used in different water matrices without interference from potential inhibitors such as detergents or surfactants commonly found in municipal wastewater [18]. Hence, close attention needs to be paid in the primer design to successfully detect specific microorganisms.

Additionally, upcoming water reuse concepts for agricultural purposes use different technologies in their water treatment train to reduce microbial loads to a minimum or completely. Hence, in order to develop new disinfection procedures and validate existing systems, monitoring procedures and knowledge of their disinfection effectiveness need to be regularly checked, especially with regard to human and plant pathogens. Thus, the main goal of this work is to develop a PCR-based detection system for relevant human DNA and plant pathogens in a hydroponic system using treated wastewater that can be used as a basis for other molecular methods. To achieve this, two approaches were considered toward this goal. First, unbiased NGS from a wastewater pond, with a specific focus on analyzing the broad variety of microorganisms within the pond and, specifically, to identify harmful human and plant pathogens, was established. Using this information, primers tailored to the relevant identified microorganisms (with respect to quantities found in the NGS and biological importance) were then created. Finally, the designed primers were tested for their effectiveness, specificity, and potential to be used as a monitoring tool for wastewater pathogens for agricultural use.

## 2. Materials and Methods

### 2.1. Water Sample Collection

The wastewater from Weißenberge, a district of Wahrenholz, Germany, with a population of around 500, is currently discharged into a treatment pond system with a surface area of approximately 4600 m^2^. No industrial or medical wastewater is discharged into these treatment ponds. The annual volume of wastewater is around 26,400 m^3^. The pond system is divided into three ponds, the first of which is a sedimentation pond and the second and third of which are aerated. The effluent of the second pond is intended to be used for hydroponic crop cultivation, as macronutrient concentrations for crop cultivation were highest. For hydroponic crop cultivation, a system based on rock wool Bato gutters with drip irrigation was selected. Before irrigation, the effluent is treated with a cascade of disinfection technologies including sand filtration, a membrane bioreactor, and UV disinfection to specifically reduce microbial load. The sampling location was selected from the effluent pipe of pond two before entering the treatment train. Samples for genome sequencing were collected on October 16, 2023, from the effluent of the second pond. Confirmation of primers as well as spiking experiments were performed with samples collected from the same location on May 13, 2024. Samples were collected and stored at −20°C until analysis. As no human or animal subjects were involved, no ethical concerns arise and thus no additional approvals were required.

### 2.2. NGS of Wastewater/Pond Sample

One hundred milliliters of each sample was centrifuged at 4300 × g for 10 min at 4°C. The supernatant was discarded, and the resulting pellet was resuspended in 1.5 mL of nuclease-free water (Invitrogen, Waltham, United States). For DNA isolation, 1 mL of the resuspended pellet was transferred to the ZR BashingBead Lysis Tubes (0.1 and 0.5 mm) (Zymo Research, Freiburg, Germany) and stored on ice immediately. Mechanical disruption was performed with a tissue homogenizer (Precellys 24, Bertin Technologies, Montigny-le-Bretonneux, France), for two times 30 s at 4000 rpm, and tubes were immediately stored on ice again in between and after mechanical disruption. After centrifugation for 10 min at 10,000 × g and 4°C, 1 mL of the supernatant was used for DNA isolation using a ZymoBiomics DNA Microprep Kit according to the manufacturer's advice (Zymo Research, Freiburg, Germany), with an elution volume of 100 *μ*L nuclease-free water. The quality of the isolated DNA was checked by the Fragment Analyzer instrument with the DNF-488 high sensitivity (HS) gDNA kit. Concentration was measured by fluorometry (Qubit 3 Fluorometer, Invitrogen) with the dsDNA HS Assay kit (Invitrogen, Waltham, United States).

Isolated nucleic acids were used for library preparation. For DNA, the Nextera XT kit (Illumina, San Diego, United States) was used according to the manufacturer's advice on the Biomek FXP pipetting robot (Beckman Coulter, Pasadena, United States) with an input of 10 ng. If the input amount was not reached, a volume of 5 *μ*L was chosen, reflecting the highest possible volume as input. The resulting DNA library was quality controlled and quantified with the DNF-474 HS NGS Kit (Fragment Analyzer Automated CE, Agilent Technologies) and Qubit 3.0 fluorometer dsDNA HS assay kit (Invitrogen, Waltham, United States). The sequencing library was prepared for sequencing by the manufacturer's protocol. In short, 5 *μ*L of the library was adjusted to 1 nM with nuclease-free water and denatured with 5 *μ*L of 0.1 M NaOH. After the addition of 5 *μ*L TRIS-HCl, the treated library was adjusted with prechilled hybridization buffer to a final loading concentration of 1.4 pM. Sequencing was performed on the MiniSeq device with the MiniSeq Rapid Kit using 100 bp single-read settings (Illumina, San Diego, United States) with a targeted sequencing depth of 20 mio reads.

### 2.3. Next-Generation Computational Pipeline Organization

Demultiplexing of sequencing data was performed using bcl2fastq (v2.20.0.422). Quality control (QC) of raw and trimmed reads was assessed with FastQC (v0.12.1). Reads were trimmed for quality, and adapters were removed using BBDuk from the BBMap package (v39.01) with the parameters trimpolyg = 10 hdist = 1 mink = 12 threads = 20 maxns = 10 minlen = 50 trimq = 20 qtrim = t ktrim = r k = 28.

Reads were mapped to the human reference genome (GRCh38) using NextGenMap (v0.5.5), and unmapped reads were extracted using samtools (v1.6). Non-human reads in BAM format were converted back to FASTQ format using bamtools (v2.5.1). Low-complexity reads were removed using PRINSEQ-lite (v0.20.4). Taxonomic classification of non-human DNA reads was performed using analysis tools (Kraken2 with the k2_eupathdb48_20230407 database from the EuPathDB project), while the classification of RNA reads was performed using a locally built full reference database, which includes sequences from RNA viruses as well. Species classifications were retained if they met a stringency threshold of 0.6 or higher, while for genus, a lower stringency threshold of 0.2 was used. Postprocessing of classified data was carried out using custom Python scripts. Results were visualized using custom R scripts to provide species-level identification and comparative analyses across samples. To support reproducibility, raw sequencing data have been deposited in the NCBI Sequence Read Archive under accession code PRJNA1248734. Primer sequences, target genes, and BLAST validation results are provided in Tables S1 and S2.

### 2.4. Specific Primer Design for PCR

Candidate primer pairs targeting unique, conserved regions of pathogen-specific genes were designed using Primer-BLAST, referencing the NCBI nucleotide database (see Table S1). Primers were evaluated based on melting temperature (Tm), GC content, absence of secondary structures, and amplicon size (target: 100–300 bp). Specificity was tested in silico using NCBI Primer-BLAST and was confirmed by alignment against the metagenomic sequences to avoid cross-reactivity with nontarget microorganisms (see Table S2 for Primer-BLAST results). Specifically, key parameter settings were as follows: product size: 70–200; product Tm: 75–90; primer size: 18–25; GC content: 30–60; and Primer Tm: 59–65; other parameters were applied at default settings. The specificity of the primer pairs was assessed in silico by conducting BLAST searches against the NCBI nucleotide database. The oligonucleotides were further analyzed for secondary structures by the Beacon Designer software (Version 8.21; [22]). Primers with no or few dimers or hairpin structures and the highest rating score were selected. All primers were synthesized by Integrated DNA Technologies.

### 2.5. Cultivation of Relevant Microorganisms

Relevant microorganisms were selected based on NGS, as described in the Results section, and were grown on standard NA, TSB, and TSY agar and medium that were prepared according to the manufacturer's instructions. All microorganisms were cultivated under shaking (170 rpm) aerobic conditions at 30°C. All relevant strains have been purchased from the DSMZ using the associated DSM numbers. All bacterial strains used in this study are listed in Table 1, along with details of media and cultivation conditions.

### 2.6. DNA Isolation and PCR Conditions

Genomic DNA from all used stains was isolated with the innuPREP Bacteria DNA Kit (IST Innuscreen GmbH), according to the manufacturer's instructions from a 1 mL overnight culture inoculated with a single colony. The concentration of DNA was measured using a NanoDrop. Extracted DNA was diluted to a concentration of 1 ng/*μ*L and stored at −20°. The extracted DNA from all bacterial strains was used for PCR amplification. To validate the designed primers, endpoint PCR was conducted using a Bio-Rad T100 Thermal Cycler. PCR reactions (25 *μ*L total volume) contained PCR Master Mix (2x) (Thermo Scientific), 0.5 *μ*M of each primer, 5 ng of metagenomic DNA, and nuclease-free water up to a final volume of 25 *μ*L. Thermocycling conditions were optimized for all primer sets to allow simultaneous amplification that included an initial denaturation at 95°C for 2 min, followed by 30 cycles of 95°C for 30 s, 55°C for 30 s, and 72°C for 30 s, with a final extension at 72°C for 10 min. Amplified products were separated on 2% agarose gels stained with Midori green DNA staining (1 *μ*L/10 mL agarose gel). Positive amplification confirmed the presence of target pathogens in the samples.

### 2.7. Sample Spiking of Collected Untreated Wastewater

To evaluate the efficiency of DNA extraction as well as the functionality of the primers in a wastewater pond matrix, a targeted spiking of the wastewater pond sample with liquid cultures of the pathogens was carried out before DNA extraction. Here, individual microorganisms were cultured in medium as previously described. Then, samples were centrifuged, and the supernatant was removed and washed with PBS buffer. Then, the samples were diluted with pond water (sterile filtered with a 0.22 *μ*m filter) collected on May 13, 2024, for a targeted concentration of a pooled sample of 1 × 10^4^ CFU/mL. Final concentrations were determined through plating on species-specific agar plates. Final bacterial loads of a plant pathogen sample were 1.5 × 10^4^ CFU/mL for *Xanthomonas vesicatoria*, 2.2 × 10^4^ CFU/mL for *Pseudomonas viridiflava*, 1.7 × 10^4^ CFU/mL for *Pseudomonas syringae*, and 3.5 × 10^4^ CFU/mL for *Rhodococcus fascians*. Identically, the five human pathogen species were added to another wastewater sample for a final bacterial load of 1.4 × 10^4^ CFU/mL for *Streptococcus mutans*, 9.9 × 10^5^ CFU/mL for *P. aeruginosa*, 4.5 × 10^5^ CFU/mL for *Acinetobacter baumannii*, 5.8 × 10^4^ CFU/mL for *Yersinia enterocolitica*, and 2.8 × 10^4^ CFU/mL for *Enterococcus faecalis*. Samples then followed DNA extraction and PCR amplification as described before.

## 3. Results

### 3.1. Metagenomic Analysis and Pathogen Identification

Raw data of the metagenomic analysis can be found on the NCBI website under Accession Code PRJNA1248734. Shotgun metagenomic sequencing generated 21,704,070 raw sequences, providing a comprehensive view of the sample's microbial diversity. After post quality filtering (Q20 threshold), 19,515,378 high-quality reads were retained. The quality-based trimming does not introduce any biases. It removes all fragmented shorter than 50 bases after adapter trimming and all low-quality reads with a Phred-score below or equal to 20. Host-derived sequences were then removed by mapping to the human genome (hg38), resulting in 16,164,297 non-host reads. The second step, metagenomic analysis of unmapped reads was performed to classify the reads and provide them with taxonomy identification. The reads were assigned to genus and species with 0.6 confidence score using Kraken2, respectively (0.6 dataset). In Kraken2, the classification confidence score is a measure of how confidently the algorithm assigns a taxonomic label to a read. The specified confidence score 0.6 means that at least 60% of the *k*-mers in a given read must support a classification to a given taxonomic ID for Kraken2 to report it. A score of 0.6 allows for a higher specificity, removing potential false-negative classification but in turn reducing the sensitivity. Correspondingly, out of a total of 16,164,297 non-human reads, 8.1% were assigned to a genus and 3.8% to a species with a high specificity.

**Figure 1:**
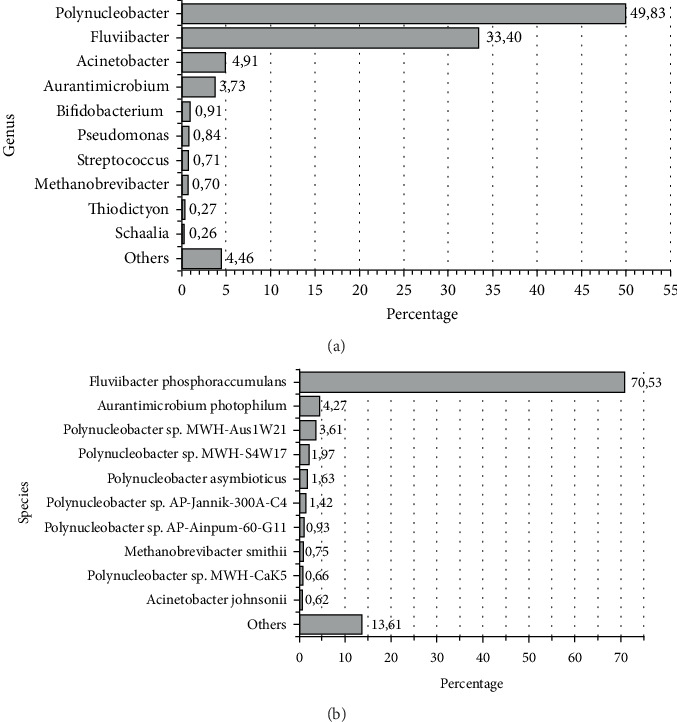
Metagenomic analysis and assignment of reads to (a) genus level and (b) species level.

The bar plots in Figure 1 show the frequency of the Top 10 classified genera/species (Figure 1a,b) classified with the classification threshold set to a confidence score equal to 0.6. *Polynucleobacter* spp. and *Fluviibacter* spp. were the most frequently occurring genera, accounting for approximately 50% and 35% of the classified genera, respectively, and *Pseudomonas* and *Streptococcus* were also among the Top 10 most frequent genera. At approximately 70%, *Fluviibacter phosphoraccumulans* was the most frequently occurring species. This is no surprise as *F. phosphoraccumulans* is a freshwater bacterioplankton, which is detected in riverine environments with high organic matter content. Moreover, five of the 10 most frequent species were *Polynucleobacter* spp. species. These bacteria can be found in aquatic environments, including wetlands, and are known to play a role in the nitrogen cycle and other microbial processes in these ecosystems.

**Figure 2:**
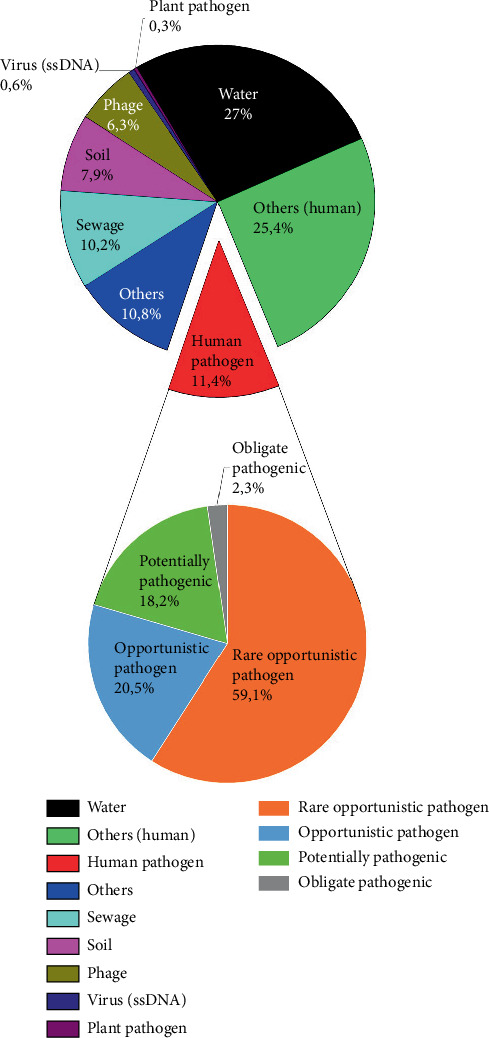
Distribution of identified species (above a threshold of 25 reads) based on their origin. Additionally, human pathogens were further categorized by their pathogenicity.

For further analysis, 1923 species were identified in the 0.6 dataset. To obtain a comprehensive overview of the species present in the sample, the detected species were categorized into nine groups, namely, water, soil, sewage, phage, virus, human pathogen, plant pathogen, others (human), and others. The group “others (human)” was designated to include species that are part of the human microbiome. The distribution of this categorization, which included the first 315 species (threshold set to ≥ 25 reads to ensure classification reliability), is illustrated in Figure 2. The largest proportion consisted of bacteria originating from water at 27%, followed by bacteria classified as part of the human microbiome (“others (human)”: 25.4%). Human pathogens formed the third largest group, accounting for 11.4%. Only one plant pathogen, *R. fascians*, could be identified (with a threshold of 25 read counts), which accounted for 0.3%, constituting the smallest proportion of classified taxa in this subset.

To further identify more plant pathogens in the sequencing sample, the 0.6 dataset was examined for known plant pathogens based on literature. A total of six known plant pathogens were identified with read counts above 4 in brackets (*P. syringae* with a lower read count was included in the consideration as it shows pathogenic behavior toward tomatoes that are cultivated in the greenhouse): *R. fascians* (69), *Acidovorax avenae* (6), *X. vesicatoria* (5), *P. viridiflava* (5), *Xanthomonas hyacinthi* (4), and *P. syringae* (1) [23–28]. Attention was given to selecting pathogens with a high read count and a preference for the host plants tomato or lettuce, as these are the primary crops cultivated with the treated water. Pathogen selection was based on a combination of metagenomic read abundance, relevance to hydroponic crop safety, and literature-reported pathogenicity. Human pathogens were prioritized if they were opportunistic or obligate pathogens with known persistence in water systems and potential human health risks. Plant pathogens were selected based on host specificity (e.g., tomato and lettuce), relevance to hydroponic crops, and previous reports of occurrence in recycled water systems. This dual approach ensures that both human health and crop productivity risks are addressed.  Table 2 lists the identified and selected human and plant pathogens along with their reference number at DSMZ, the host plants, and the read count from the sequencing.

In order to select the most relevant human pathogens for a PCR detection test system, the “human pathogen” group, containing 44 species (above the threshold of 25 reads), was further categorized into four subgroups: obligate pathogens, opportunistic pathogens, rare opportunistic pathogens, and potentially pathogenic species.

Figure 2 (lower chart) illustrates the distribution of these groups. Notably, the rare opportunistic pathogens represented the largest proportion at 59.1%, followed by opportunistic pathogens at 20.5%, and potentially pathogenic species at 18.2%. Interestingly, among all identified human pathogens, only one (2.3%) obligate pathogen was found. Based on metagenomic analysis, the human pathogens listed in Table 2, namely, *S. mutans*, *P. aeruginosa*, *A. baumannii*, *Y. enterocolitica*, and *E. faecalis*, were chosen for the PCR detection test system based on their read count and identified pathogenicity described in the literature and the highest read counts in the NGS [28, 33–36].

### 3.2. Primer Design for Human and Plant Pathogen Species

The primer design process resulted in primer sets listed in Table 3, along with their corresponding expected amplicon size. Key parameters such as primer length, GC content, Tm, and potential secondary structures were optimized, as described in the Materials and Methods section, to ensure both efficient and specific amplification. In silico analysis revealed high specificity of the designed primers toward the target sequence. BLAST searches against public databases further validated the primers' species specificity (see Table S2).

**Figure 3:**
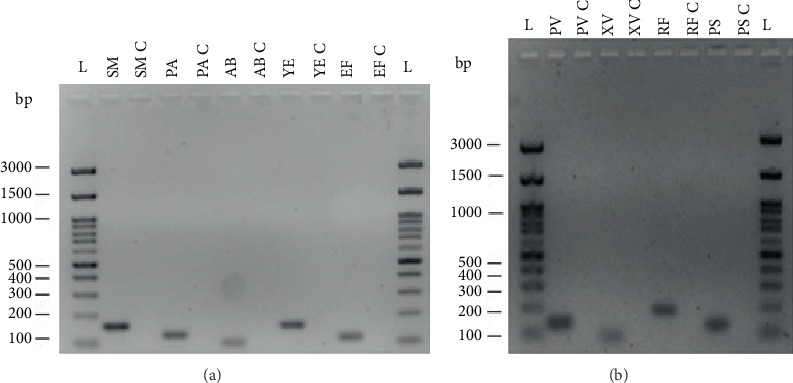
Gel electrophoresis images of PCR products. (a) Image of fragments of human pathogens and (b) image of fragments of plant pathogens. Abbreviations: bp, base pair; L, FastGene 100 bp DNA ladder; C, negative control without any DNA; *SM*, *Streptococcus mutans*; *PA*, *Pseudomonas aeruginosa*; *AB*, *Acinetobacter baumannii*; *YE*, *Yersinia enterocolitica*; *EF*, *Enterococcus faecalis*; *PV*, *Pseudomonas viridiflava*; *XV*, *Xanthomonas vesicatoria*; *RF*, *Rhodococcus fascians*; *PS*, *Pseudomonas syringae*.

The designed primer pairs were tested through PCR amplification using genomic DNA from the target microorganisms as described in the Materials and Methods section. Gel electrophoresis of the PCR products (Figure 3) confirmed that the primer pairs for detection of their target species produced a single, distinct band at the expected fragment size (see Table 3 for fragment length) only in the presence of the target DNA. Nevertheless, band color intensities vary possibly as a result of different gDNA concentrations after extraction. These results confirm the specificity of each primer pair and validate the expected amplicon lengths (Table 3).

**Figure 4:**
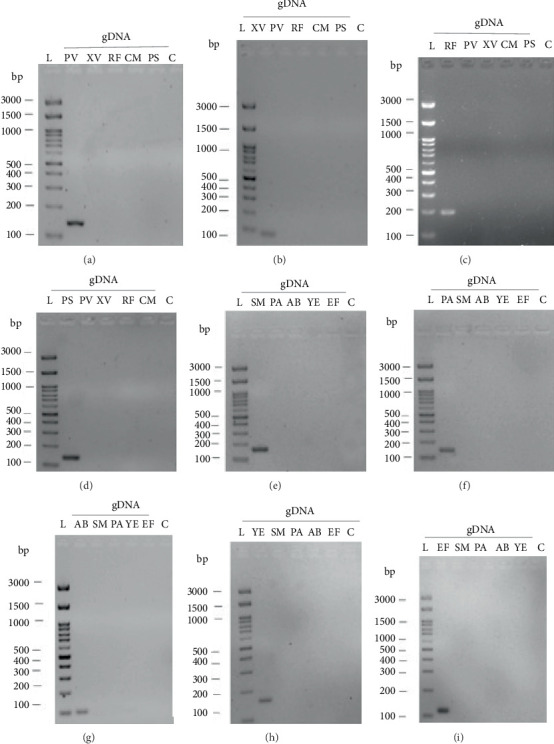
Gel electrophoresis images of PCR products for cross contamination check with microorganisms used in this work. The individual images show cross contaminations checks containing (a) gDNA from *Pseudomonas viridiflava* and individual specific primer pairs, (b) gDNA from *Xanthomonas vesicatoria* and individual specific primer pairs, (c) gDNA from *Rhodococcus fascians* and individual specific primer pairs, (d) gDNA from *Pseudomonas syringae* and individual specific primer pairs, (e) gDNA from *Streptococcus mutans* and individual specific primer pairs, (f) gDNA from *Pseudomonas viridiflava* and individual specific primer pairs, (g) gDNA from *Acinetobacter baumannii* and individual specific primer pairs, (h) gDNA from *Yersinia enterocolitica* and individual specific primer pairs, and (i) gDNA from *Enterococcus faecalis* and individual specific primer pairs. Abbreviations: bp, base pair; L, FastGene 100 bp DNA ladder; C, negative control without any DNA; *SM*, *Streptococcus mutans*; *PA*, *Pseudomonas aeruginosa*; *AB*, *Acinetobacter baumannii*; *YE*, *Yersinia enterocolitica*; *EF*, *Enterococcus faecalis*; *PV*, *Pseudomonas viridiflava*; *XV*, *Xanthomonas vesicatoria*; *RF*, *Rhodococcus fascians*; *PS*, *Pseudomonas syringae*.

To further validate the specificity of the designed primers, a cross-validation of the primers was performed (Figure 4). Here, the primer pair from one species was tested against the extracted gDNA of each of the other four human pathogens or three plant pathogens to assess potential cross-reaction that would limit the specificity of the designed primers. The gels confirm that no cross-reaction with the other primers considered in this work takes place, thus confirming that each primer pair is species-specific and can be used for further analysis. Hence, no cross-reactivity was observed, confirming that each primer pair exclusively amplifies its target species, thereby minimizing the risk of false positives in environmental samples.

**Figure 5:**
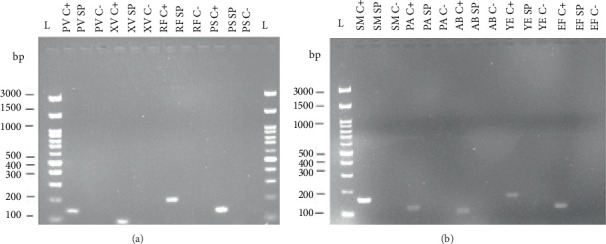
Gel electrophoresis images of DNA extractions from wastewater pond samples. (a) Primer pairs to test for specific plant pathogens. (b) Primer pairs to test for specific human pathogens. Abbreviations: bp, base pair; L, FastGene 100 bp DNA ladder; C+, positive control containing respective extracted DNA; C−, negative control without any DNA; SP, sample with extracted DNA from the sewage pond; *SM*, *Streptococcus mutans*; *PA*, *Pseudomonas aeruginosa*; *AB*, *Acinetobacter baumannii*; *YE*, *Yersinia enterocolitica*; *EF*, *Enterococcus faecalis*; *PV*, *Pseudomonas viridiflava*; *XV*, *Xanthomonas vesicatoria*; *RF*, *Rhodococcus fascians*; *PS*, *Pseudomonas syringae*.

As part of the verification of the primer pairs on a wastewater pond sample, DNA was extracted from the wastewater pond sample collected on May 13, 2024, followed by PCR. Here, the extracted DNA concentration was 2 ng/*μ*L using NanoDrop spectrophotometry. The result of the agarose gel electrophoresis is shown in Figure 5.

The positive controls (C+) contained identical samples as used for the experiments of Figure 4, while sewage pond (SP) samples contained extracted DNA from the SP as well as the respective primer pairs of interest. The negative controls (C−) contained nuclease-free water instead of DNA and served as contamination controls. The results in Figure 5 show that all positive controls for the tested primers display a band of the expected size. In contrast, no bands are visible in the setups with the wastewater pond sample or in the negative controls. Target DNA levels were below the detection limit in the unspiked wastewater sample.

To evaluate the efficiency of DNA extraction as well as the functionality of the primers in a wastewater pond matrix, a targeted spiking of the wastewater pond sample with liquid cultures of the pathogens was carried out before DNA extraction as described before. DNA extraction from the spiked SP sample showed a concentration of 87 ng/*μ*L (plant pathogen sample) and 210 ng/*μ*L (human pathogen sample) and thus confirmed a successful DNA extraction procedure. Increased DNA concentrations are the result of high concentrations of CFU for *S. mutans* and *P. aeruginosa*.

**Figure 6:**
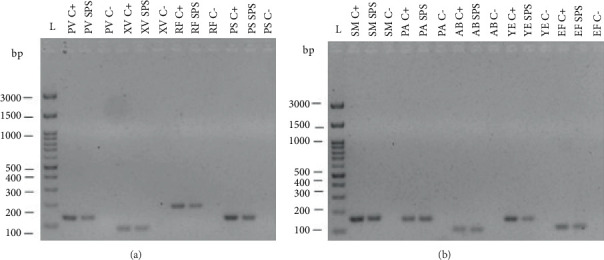
Gel electrophoresis images of DNA extractions from wastewater pond samples spiked with the species of interest. (a) Primer pairs to test for specific plant pathogens. (b) Primer pairs to test for specific human pathogens. Abbreviations: bp, base pair; L, FastGene 100 bp DNA ladder; C+, positive control containing respective extracted DNA; C−, negative control without any DNA; SPS, spiked sample with extracted DNA from the sewage pond; *SM*, *Streptococcus mutans*; *PA*, *Pseudomonas aeruginosa*; *AB*, *Acinetobacter baumannii*; *YE*, *Yersinia enterocolitica*; *EF*, *Enterococcus faecalis*; *PV*, *Pseudomonas viridiflava*; *XV*, *Xanthomonas vesicatoria*, *RF*, *Rhodococcus fascians*; *PS*, *Pseudomonas syringae*.

The result of the agarose gel electrophoresis is shown in Figure 6. The positive controls (C+) contained the reaction mixture of the respective primer pairs along with the corresponding target DNA and were used to verify the functionality of the PCR setup. In the setups containing the spiked and extracted wastewater pond sample (SPS), the effectiveness of the primers in the target organism-enriched wastewater pond matrix was tested. The negative controls (C−) contained nuclease-free water instead of DNA and were used to check for contamination. The results show that all positive controls display clearly visible bands of the expected size. Bands of the expected size are also visible for all considered species when spiked with bacterial species before the extraction. These results demonstrate the PCR system's effectiveness in detecting both human and plant pathogens in complex wastewater matrices at concentrations ≥ 10^4^ CFU/mL.

## 4. Discussion

The primary objective of this study was to identify human and plant DNA pathogens from a wastewater treatment pond system and to design specific primer pairs for their detection via PCR. Pathogen detection is critical when considering the reuse of treated wastewater for hydroponic crop cultivation. The metagenomic analysis revealed that the most abundant genera in the wastewater pond DNA sample were *Polynucleobacter* spp. and *Fluviibacter* spp.—typical bacterioplankton in freshwater systems such as lakes and ponds [37, 38]. Other abundant genera included *Acinetobacter* spp., *Pseudomonas* spp., and *Streptococcus* spp., which align with previous studies on microbial communities in wastewater and hydroponic environments [6, 17].

Of the 315 species identified, human pathogens made up 11.4%, ranking as the third-largest group. Among the five selected for further investigation, *Yersinia* spp. and *Pseudomonas* spp. are well-established wastewater-associated pathogens [8], especially *P. aeruginosa*—known for robust biofilm formation that supports persistence on surfaces such as tubing and reservoirs [39]. *Yersinia* spp. species, including *Y. enterocolitica*, are typically introduced from external sources such as untreated wastewater or contaminated equipment and have been detected in various aquatic environments [8]. Recirculating hydroponic systems pose a higher risk for pathogen survival and spread due to water reuse and biofilm development thus posing an increased risk to human health for workers or when disposed of on consumed crops. Similarly, *A. baumannii* and *P. aeruginosa* are part of the ESKAPE group—multidrug-resistant organisms known for causing severe nosocomial infections, particularly in immunocompromised individuals [40]. These findings emphasize the importance of pathogen surveillance in wastewater used for food production systems.

The metagenomic findings in this work coincide with previously published work. For instance, Azli et al. [41] detected similar species of human pathogens, as shown in this work (plant pathogens were not considered), such as *Enterococcus* spp., *A. baumannii*, or *P. aeruginosa*. Further, their analysis shows a similar class distribution of a municipal wastewater treatment plant effluent as seen here. To the best of our knowledge, this work also considered potential risks of bacterial plant pathogens in treated wastewater that can have an influence on crop cultivation for the first time. Nevertheless, *R. fascians*, which was found in the highest abundance in our analysis, has been previously described in rockwool in circulating nutrient solutions for hydroponic systems [42].

Among the 315 categorized species, *R. fascians* was the only plant pathogen identified with read counts higher than 25 (read count: 69) with five more plant pathogens identified with lower read counts than 25 (when a threshold of 25 read counts is ignored) (Figure 2). Given this, the entire dataset was screened for additional known plant pathogens. Literature-based selection yielded several important pathogens: *R. fascians*, known to cause growth abnormalities [29]; *P. viridiflava*, associated with soft rot in crops like tomatoes and lettuce [30]; *X. vesicatoria*, the causal agent of bacterial spot in tomatoes and peppers [31]; and *P. syringae*, a widespread pathogen of agricultural crops [32].

Despite this targeted screening, only six plant pathogens were identified, all with low read counts (1–69), indicating a low relative abundance of plant pathogen DNA. This low concentration is likely due to the origin of municipal wastewater, which typically comprises household effluents (e.g., kitchen, laundry, and fecal waste) and lacks agricultural inputs [43]. In contrast, agricultural wastewater is more likely to contain plant pathogens due to inputs from livestock, poultry, and crop production [44].

The specificity and functionality of the designed primers were validated using endpoint PCR with extracted DNA from target organisms. All reactions produced bands at the expected sizes (Figure 3), and no cross-reactions were observed with nontarget DNA (Figure 4), confirming high primer specificity and potential applicability in multiplex PCR.

However, when the primers were tested on DNA extracted directly from the wastewater pond sample, no bands were observed (Figure 5). Despite successful amplification in spiked samples, the absence of bands in untreated wastewater samples suggests potential PCR inhibition or insufficient DNA concentration. Wastewater matrices often contain inhibitors such as humic acids, surfactants, and heavy metals that interfere with DNA polymerase activity. Future experiments should include inhibitor-resistant polymerases, dilution series, and purification steps (e.g., inhibitor removal kits or column-based clean-up) to assess and mitigate inhibition. Additionally, preconcentration methods such as ultrafiltration or membrane filtration should be optimized to increase DNA yield from environmental samples. Positive control reactions using the same matrix but spiked with cultured pathogens confirmed that the PCR assay itself was functional. These findings suggest either that the concentration of target DNA in the environmental sample was too low, or that PCR inhibition occurred due to interfering substances in the wastewater matrix [45]. At this point, no experimental verification for PCR inhibitors as well as inhibitor-resistant DNA polymerases or additional purification steps (e.g., dilution and inhibitor removal kits) was considered. These steps could improve the detection of species, especially in environmental samples with a challenging water matrix as shown by Schrader et al. [46]. Hence, it is unknown whether the identification of microorganisms in pond samples was associated with inhibitor factors.

The total DNA concentration in the sample was only 2 ng/*μ*L, and the low read counts from sequencing further support the conclusion that the abundance of target organisms was minimal [47]. A known challenge in detecting microorganisms in water and wastewater samples is the efficient extraction and concentration of microbial DNA. Low extraction efficiency can significantly impair PCR sensitivity [20]. To address this, preextraction concentration methods such as ultrafiltration should be considered, as this technique has proven effective for recovering microorganisms from large-volume samples [48]. Additionally, the use of specialized DNA extraction kits for environmental samples, such as water or soil, may improve yield and purity [49]. Other techniques that could improve the detection of microorganisms also include centrifuging larger volumes, pooling pellets, or filtering samples through 0.2 *μ*m membranes to concentrate microorganisms, which should specifically be applied to environmental samples. Optimization of extraction protocols could allow for the detection of a broader range of pathogens, including those present at low abundance.

To assess the performance of the primers in a realistic matrix, pond water samples were spiked with liquid cultures of the target organisms prior to extraction. Gel electrophoresis results for these spiked samples (SPS) and positive controls showed successful amplification (Figure 6). While positive controls yielded strong bands, the SPS samples exhibited weaker signal intensities. This reduction could be attributed to impurities and lower DNA quality in the wastewater-derived samples, as well as the inherently lower concentration of spiked organisms. These factors are known to reduce PCR efficiency [45, 49]. Nevertheless, the results confirmed that the primer pairs can detect target DNA in complex wastewater matrices when present above a certain threshold. While endpoint PCR confirmed primer specificity, qPCR and dPCR offer enhanced sensitivity and allow for precise quantification of pathogen DNA. These methods are particularly valuable for environmental samples with low microbial loads and complex matrices. Future work should integrate qPCR workflows using the validated primers to establish detection thresholds, assess treatment efficacy, and monitor pathogen dynamics over time. This would also enable compliance with regulatory frameworks that require quantitative microbial risk assessments.

## 5. Conclusions

This study provides novel insights into the microbial composition of municipal wastewater and identifies relevant human and plant pathogens with implications for safe reuse in hydroponic crop cultivation. Specific primer pairs were successfully designed and validated for PCR-based detection of these pathogens. Specifically, the designed primers are intended to support high-throughput sample analysis to screen for potential human and plant pathogens without the requirement of expensive and time-consuming analyses such as NGS. This will also allow faster feedback on microbial water quality and potential risk factors entering a production site. Similarly, the designed primers will help to evaluate the success of water treatment by selecting sampling sites before and after treatment when establishing robust concentration methods, such as ultrafiltration, prior to DNA extraction, which will be essential for ensuring sufficient DNA input for reliable detection [48].

Future research should focus on integrating these primers into RT-qPCR workflows to enhance sensitivity and enable quantification of pathogen DNA [50, 51]. Sampling at multiple time points may offer further insights into the temporal dynamics of pathogen presence in wastewater. Additionally, including RNA analyses would allow for the detection of important RNA viruses—both human- and plant-derived—such as *Comamonas testosteroni* [52] and tomato brown rugose fruit virus [53], which may impact human health or crop productivity if introduced via hydroponic systems.

## Figures and Tables

**Table 1:** Bacteria strains, media, and cultivation conditions used for this study.

**Species**	**DSM number**	**Cultivation medium**	**Cultivation conditions**
*Streptococcus mutans*	20523	TSY	37°C; 1–2 days
*Pseudomonas aeruginosa*	1128	TSY	37°C; 1–2 days
*Acinetobacter baumannii*	30007	TSY	30°C; 1–2 days
*Yersinia enterocolitica*	4780	TSY	30°C; 1–2 days
*Enterococcus faecalis*	20478	TSY	37°C; 1–2 days
*Rhodococcus fascians*	20669	TSB	29°C; 1–2 days
*Pseudomonas viridiflava*	6694	NA	29°C; 1–2 days
*Xanthomonas vesicatoria*	22252	NA	29°C; 3–7 days
*Clavibacter michiganensis*	46364	TSY	29°C; 3–7 days
*Pseudomonas syringae*	6693	NA	29°C; 1–2 days

**Table 2:** Human and plant pathogens selected for the PCR/qPCR detection test system due to the highest read counts from the next-generation sequencing.

**Pathogen type**	**Pathogen species**	**DSMZ #**	**Subgroup**	**Read count**
Human	*Streptococcus mutans*	20523	Opportunistic pathogen	223
	*Pseudomonas aeruginosa*	1128	Opportunistic pathogen	174
	*Acinetobacter baumannii*	30007	Opportunistic pathogen	172
	*Yersinia enterocolitica*	4780	Obligate pathogen	62
	*Enterococcus faecalis*	20478	Opportunistic pathogen	32

Plant	*Rhodococcus fascians*	20669	Tobacco and thale cress [29]	69
	*Pseudomonas viridiflava*	6694	Tomato, lettuce, and eggplant [30]	5
	*Xanthomonas vesicatoria*	22252	Tomato and pepper [31]	5
	*Pseudomonas syringae*	6693	Tomato, cabbage, and cucumber [32]	1

**Table 3:** Species-specific primers for PCR identification of human and plant pathogens found in the sample from Wahrenholz.

**Pathogen type**	**Pathogen species**	**Target gene**	**Primer**	**Sequence (5 **⁣′** –3 **⁣′**)**	**Product size (bp)**
Human	*Streptococcus mutans* (*SM*)	gtfC	Forward Reverse	ACAGATGCTGCAAACTTCGAACA CGCTGCGTTTCTTGGTCAGG	161
	*Pseudomonas aeruginosa* (*PA*)	16S	Forward Reverse	TATGAAGGGAGCTTGCCTTGGA TAGCGTGAGGTCCGAAGATCC	148
	*Acinetobacter baumannii* (*AB*)	rpoB	Forward Reverse	GATCACGCGTCAAGTCAGCA TGGCAGAAGGCGGTGTTAAG	102
	*Yersinia enterocolitica* (*YE*)	inv	Forward Reverse	GGTGCAGAAGCCTGGACTGA CACCCAACTGTGGAAGTGCAG	163
	*Enterococcus faecalis* (*EF*)	sodA	Forward Reverse	TGGACAACCAACTGGCGCTAT CCAAGCCCAACCTGAACCAA	118

Plant	*Pseudomonas viridiflava* (*PV*)	gyrB	Forward Reverse	CGTAGGCGAGAGCGATACCA ATGATGCCGACGCCAGAGTT	141
	*Xanthomonas vesicatoria* (*XV*)	hrpB	Forward Reverse	GTGCTGTCTCTGCGGGAATG GGCGTAGCCAATGGTCCAAC	91
	*Rhodococcus fascians* (*RF*)	rsmA	Forward Reverse	TCGTTGACTCTCGCTCTGCT CACCAATGCCGTCGGGTATC	192
	*Pseudomonas syringae* (*PS*)	hrpL	Forward Reverse	AGTTTCAGCACGCCAGCAAA CGTTCGACTCCAGGTCCGTA	141

## Data Availability

All data supporting the findings of this study are included within the manuscript and its supporting information. Further, all data from the NGS analysis are available in the NCBI public repository under BioSample: SAMN47867322.

## Nomenclature

AB: *Acinetobacter baumannii*
BAM: binary alignment/map format
BLAST: basic local alignment search tool
CFUs: colony forming units
DNA: deoxyribonucleic acid
DSM: Deutsche Sammlung von Mikroorganismen
DSMZ: German Collection of Microorganisms and Cell Cultures
EF: *Enterococcus faecalis*
ESKAPE: *Enterococcus*, *Staphylococcus*, *Klebsiella*, *Acinetobacter*, *Pseudomonas*, *Enterobacter*
FASTQ: file format for storing sequencing data
GC: guanine–cytosine content
ID: identification
MPMI: molecular plant–microbe interactions
NCBI: National Center for Biotechnology Information
NGS: next-generation sequencing
PA: *Pseudomonas aeruginosa*
PBS: phosphate buffered saline
PCR: polymerase chain reaction
PS: *Pseudomonas syringae*
PV: *Pseudomonas viridiflava*
QC: quality control
RF: *Rhodococcus fascians*
RNA: ribonucleic acid
RT: reverse transcription
SM: *Streptococcus mutans*
SP: sewage pond
SPS: spiked pond sample
TSB: tryptic soy broth
TSY: tryptic soy yeast
UV: ultraviolet
VBNC: viable but nonculturable
XV: *Xanthomonas vesicatoria*
YE: *Yersinia enterocolitica*

## Supporting Information

Supporting Information Additional supporting information can be found online in the Supporting Information section. Table S1: Name of target genes with gene description and NCBI reference no. The table further specifies the specific name of the target gene, the gene description, the NCBI reference number, and the reference where it was first published for each microorganism considered. Table S2: BLAST results for each individual primer with the Top 10 results shown (except for *Enterococcus faecalis*). The list described the description of identified microorganism and scientific name, as well as BLAST specific results of identification.
