# Supplementary material for: Molecular Identification of Human and Plant Pathogens in Municipal Domestic Wastewater for Hydroponic System Applications
Source: Int J Microbiol. 2025 Nov 25;2025:6958575. doi: 10.1155/ijm/6958575 (PMC12672069; doi:10.1155/ijm/6958575)
Supplement: Supporting Information — Additional supporting information can be found online in the Supporting Information section. Table S1: Name of target genes with gene description and NCBI reference no. The table further specifies the specific name of the target gene, the gene description, the NCBI reference number, and the reference where it was first published for each microorganism considered. Table S2: BLAST results for each individual primer with the Top 10 results shown (except for Enterococcus faecalis). The list described the description of identified microorganism and scientific name, as well as BLAST specific results of identification. [file 6958575.f1.docx]

Supplementary Material

# Supplementary Table

S1: Name of target genes with gene description and NCBI reference no.

| **Pathogen species** | **Name of target gene** | **Gene description** | **NCBI Reference No.** | **References** |
| --- | --- | --- | --- | --- |
| *Streptococcus mutans* | gtfC | glucosyltransferase GtfC | NZ_CP044221.1 | (Argimón & Caufield, 2020) |
| *Pseudomonas aeruginosa* | 16S | 16S ribosomal RNA | NR_026078.1 | (de Oliveira et al., 2024) |
| *Acinetobacter baumannii* | rpoB | rpoB DNA-directed RNA polymerase subunit beta | NZ_CP045110.1 | (de Oliveira et al., 2024) |
| *Yersinia enterocolitica* | inv | inverse autotransporter invasin Inv | NC_017564.1 | (Bancerz-Kisiel et al., 2018) |
| *Enterococcus faecalis* | sodA | superoxide dismutase | NZ_KB944666.1 | (Peykov et al., 2012) |
| *Pseudomonas viridiflava* | gyrB | GyrB DNA topoisomerase (ATP-hydrolyzing) subunit B | NZ_LT855380.1 | (Bartoli et al., 2014) |
| *Xanthomonas vesicatoria* | hrpB | hrpB ATP-dependent helicase | NZ_CP018725.1 | (Zhu et al., 2000) |
| *Rhodococcus fascians* | rsmA | 6S rRNA (adenine(1518)-N(6)/adenine(1519)-N(6))-dimethyltransferase | NZ_CP015235.1 | (Jameson et al., 2019) |
| *Pseudomonas syringae* | hrpL | RNA polymerase sigma factor | NZ_CP074578.1 | (Vaseghi et al., 2014) |

**S2: BLAST results for each individual primer with the Top 10 results shown (except for *Entercococcus faecalis*)**

| ***Streptococcus mutans* (SM)** | **Forward: ACAGATGCTGCAAACTTCGAACA** | | | | |  |  |  |
| --- | --- | --- | --- | --- | --- | --- | --- | --- |
| **Description** | **Scientific Name** | **Max Score** | **Total Score** | **Query Cover** | **E value** | | **Per. ident** | **Acc. Len** |
| Streptococcus mutans strain P1 chromosome, complete genome | Streptococcus mutans | 461 | 461 | 100,00% | 23 | | 10000 | 2085371 |
| Streptococcus mutans strain P6 chromosome, complete genome | Streptococcus mutans | 461 | 461 | 100,00% | 23 | | 10000 | 2085254 |
| Streptococcus mutans strain S4 chromosome, complete genome | Streptococcus mutans | 461 | 461 | 100,00% | 23 | | 10000 | 2085365 |
| Streptococcus mutans B04Sm5 chromosome, complete genome | Streptococcus mutans B04Sm5 | 461 | 461 | 100,00% | 23 | | 10000 | 2011542 |
| Streptococcus mutans strain LAR01 chromosome, complete genome | Streptococcus mutans | 461 | 461 | 100,00% | 23 | | 10000 | 2088369 |
| Streptococcus mutans strain FDAARGOS 1458 chromosome, complete genome | Streptococcus mutans | 461 | 461 | 100,00% | 23 | | 10000 | 2019345 |
| Streptococcus mutans strain 27-3 chromosome, complete genome | Streptococcus mutans | 461 | 461 | 100,00% | 23 | | 10000 | 1978522 |
| Streptococcus mutans NBRC 13955 DNA, complete genome | Streptococcus mutans | 461 | 461 | 100,00% | 23 | | 10000 | 2018796 |
| Streptococcus mutans NN2025 DNA, complete genome | Streptococcus mutans NN2025 | 461 | 461 | 100,00% | 23 | | 10000 | 2013587 |
| Streptococcus mutans strain NCH105 chromosome, complete genome | Streptococcus mutans | 461 | 461 | 100,00% | 23 | | 10000 | 2015393 |
| ***Streptococcus mutans* (SM)** | **Reverse: CGCTGCGTTTCTTGGTCAGG** | | | |  | |  |  |
| **Description** | **Scientific Name** | **Max Score** | **Total Score** | **Query Cover** | **E value** | | **Per. ident** | **Acc. Len** |
| Streptococcus mutans strain P1 chromosome, complete genome | Streptococcus mutans | 401 | 401 | 100,00% | 14 | | 10000 | 2085371 |
| Streptococcus mutans strain P6 chromosome, complete genome | Streptococcus mutans | 401 | 401 | 100,00% | 14 | | 10000 | 2085254 |
| Streptococcus mutans strain LAB761 chromosome, complete genome | Streptococcus mutans | 401 | 401 | 100,00% | 14 | | 10000 | 2076490 |
| Streptococcus mutans strain S4 chromosome, complete genome | Streptococcus mutans | 401 | 401 | 100,00% | 14 | | 10000 | 2085365 |
| PREDICTED: Equus quagga IQ motif and ubiquitin domain containing (IQUB), transcript variant X1, mRNA | Equus quagga | 401 | 401 | 100,00% | 14 | | 10000 | 4134 |
| PREDICTED: Bactrocera dorsalis proton-coupled folate transporter (LOC105227277), transcript variant X2, mRNA | Bactrocera dorsalis | 401 | 401 | 100,00% | 14 | | 10000 | 2023 |
| Streptococcus mutans B04Sm5 chromosome, complete genome | Streptococcus mutans B04Sm5 | 401 | 401 | 100,00% | 14 | | 10000 | 2011542 |
| Streptococcus mutans strain LAR01 chromosome, complete genome | Streptococcus mutans | 401 | 401 | 100,00% | 14 | | 10000 | 2088369 |
| PREDICTED: Equus asinus IQ motif and ubiquitin domain containing (IQUB), transcript variant X1, mRNA | Equus asinus | 401 | 401 | 100,00% | 14 | | 10000 | 6877 |
| PREDICTED: Equus quagga IQ motif and ubiquitin domain containing (IQUB), transcript variant X4, mRNA | Equus quagga | 401 | 401 | 100,00% | 14 | | 10000 | 3957 |

| ***Pseudomonas aeruginosa* (PA)** | **Forward: TATGAAGGGAGCTTGCCTTGGA** | | | | | | |  |  |  |
| --- | --- | --- | --- | --- | --- | --- | --- | --- | --- | --- |
| **Description** | **Scientific Name** | | **Max Score** | | **Total Score** | | **Query Cover** | **E value** | **Per. ident** | **Acc. Len** |
| Pseudomonas aeruginosa strain Pa5 16S ribosomal RNA gene, partial sequence | Pseudomonas aeruginosa | | 441 | | 441 | | 100,00% | 89 | 10000 | 1441 |
| Pseudomonas aeruginosa strain Pa24 16S ribosomal RNA gene, partial sequence | Pseudomonas aeruginosa | | 441 | | 441 | | 100,00% | 89 | 10000 | 1465 |
| Pseudomonas aeruginosa strain EW14 16S ribosomal RNA gene, partial sequence | Pseudomonas aeruginosa | | 441 | | 441 | | 100,00% | 89 | 10000 | 1375 |
| Pseudomonas aeruginosa strain sample4 16S ribosomal RNA gene, partial sequence | Pseudomonas aeruginosa | | 441 | | 441 | | 100,00% | 89 | 10000 | 1433 |
| Pseudomonas aeruginosa strain Pa22 16S ribosomal RNA gene, partial sequence | Pseudomonas aeruginosa | | 441 | | 441 | | 100,00% | 89 | 10000 | 1448 |
| Pseudomonas aeruginosa strain SSI043 16S ribosomal RNA gene, partial sequence | Pseudomonas aeruginosa | | 441 | | 441 | | 100,00% | 89 | 10000 | 843 |
| Pseudomonas aeruginosa strain SACMBR04 16S ribosomal RNA gene, partial sequence | Pseudomonas aeruginosa | | 441 | | 441 | | 100,00% | 89 | 10000 | 1398 |
| Pseudomonas aeruginosa strain Pa20 16S ribosomal RNA gene, partial sequence | Pseudomonas aeruginosa | | 441 | | 441 | | 100,00% | 89 | 10000 | 1450 |
| Pseudomonas paraeruginosa strain PA7 16S ribosomal RNA gene, partial sequence | Pseudomonas paraeruginosa PA7 | | 441 | | 441 | | 100,00% | 89 | 10000 | 1440 |
| Pseudomonas aeruginosa strain Pa8 16S ribosomal RNA gene, partial sequence | Pseudomonas aeruginosa | | 441 | | 441 | | 100,00% | 89 | 10000 | 1450 |
| ***Pseudomonas aeruginosa* (PA)** | **Reverse: TAGCGTGAGGTCCGAAGATCC** | | | | | | |  |  |  |
| **Description** | **Scientific Name** | **Max Score** | | **Total Score** | | **Query Cover** | | **E value** | **Per. ident** | **Acc. Len** |
| Pseudomonas aeruginosa strain WYT chromosome, complete genome | Pseudomonas aeruginosa | 421 | | 168 | | 100,00% | | 35 | 10000 | 6958497 |
| Pseudomonas aeruginosa isolate 2024CK-01834 chromosome, complete genome | Pseudomonas aeruginosa | 421 | | 168 | | 100,00% | | 35 | 10000 | 6924229 |
| Pseudomonas aeruginosa strain 2021CK-01158 chromosome, complete genome | Pseudomonas aeruginosa | 421 | | 168 | | 100,00% | | 35 | 10000 | 6549656 |
| Pseudomonas aeruginosa strain NY5511 chromosome, complete genome | Pseudomonas aeruginosa | 421 | | 168 | | 100,00% | | 35 | 10000 | 6850748 |
| Pseudomonas aeruginosa strain HS_121 chromosome, complete genome | Pseudomonas aeruginosa | 421 | | 168 | | 100,00% | | 35 | 10000 | 6663921 |
| MAG: uncultured Marinobacterium sp. isolate 9dab0b5a-edc3-4719-b21f-a034148b1e2d genome assembly, chromosome: 1 | uncultured Marinobacterium sp. | 421 | | 252 | | 100,00% | | 35 | 10000 | 4113871 |
| Pseudomonas aeruginosa strain PALA40 chromosome, complete genome | Pseudomonas aeruginosa | 421 | | 168 | | 100,00% | | 35 | 10000 | 7089219 |
| Pseudomonas aeruginosa strain PAE3 chromosome, complete genome | Pseudomonas aeruginosa | 421 | | 168 | | 100,00% | | 35 | 10000 | 7073555 |
| Pseudomonas aeruginosa strain 14182 chromosome, complete genome | Pseudomonas aeruginosa | 421 | | 168 | | 100,00% | | 35 | 10000 | 7144174 |
| Pseudomonas aeruginosa strain PaLo524 chromosome, complete genome | Pseudomonas aeruginosa | 421 | | 168 | | 100,00% | | 35 | 10000 | 6459518 |

| ***Acinetobacter baumannii* (AB)** | **Forward: GATCACGCGTCAAGTCAGCA** | | | |  |  |  |
| --- | --- | --- | --- | --- | --- | --- | --- |
| **Description** | **Scientific Name** | **Max Score** | **Total Score** | **Query Cover** | **E value** | **Per. ident** | **Acc. Len** |
| Acinetobacter baumannii strain 2022CK-00066 chromosome, complete genome | Acinetobacter baumannii | 401 | 401 | 100,00% | 14 | 10000 | 4029133 |
| Acinetobacter baumannii strain A20AB02 chromosome, complete genome | Acinetobacter baumannii | 401 | 401 | 100,00% | 14 | 10000 | 3935091 |
| Acinetobacter baumannii strain E20AB39 chromosome | Acinetobacter baumannii | 401 | 401 | 100,00% | 14 | 10000 | 4023122 |
| Acinetobacter baumannii strain 2024CK-00247 chromosome, complete genome | Acinetobacter baumannii | 401 | 401 | 100,00% | 14 | 10000 | 3989019 |
| Acinetobacter baumannii strain A20AB01 chromosome, complete genome | Acinetobacter baumannii | 401 | 401 | 100,00% | 14 | 10000 | 4027555 |
| Acinetobacter baumannii strain 19WIARLN021_full chromosome, complete genome | Acinetobacter baumannii | 401 | 401 | 100,00% | 14 | 10000 | 3956311 |
| Acinetobacter baumannii strain CQPMC-AB07 chromosome, complete genome | Acinetobacter baumannii | 401 | 401 | 100,00% | 14 | 10000 | 3932098 |
| Acinetobacter baumannii strain 2023CK-01487 chromosome, complete genome | Acinetobacter baumannii | 401 | 401 | 100,00% | 14 | 10000 | 4009122 |
| Acinetobacter baumannii strain A. baumannii chromosome | Acinetobacter baumannii | 401 | 401 | 100,00% | 14 | 10000 | 3959175 |
| Acinetobacter baumannii strain SNUBHAB0291 chromosome, complete genome | Acinetobacter baumannii | 401 | 401 | 100,00% | 14 | 10000 | 3975597 |

| ***Acinetobacter baumannii* (AB)** | **Reverse: TGGCAGAAGGCGGTGTTAAG** | | | |  |  |  |
| --- | --- | --- | --- | --- | --- | --- | --- |
| **Description** | **Scientific Name** | **Max Score** | **Total Score** | **Query Cover** | **E value** | **Per. ident** | **Acc. Len** |
| Acinetobacter baumannii strain SIMBA003 chromosome, complete genome | Acinetobacter baumannii | 401 | 401 | 100,00% | 14 | 10000 | 3778202 |
| Acinetobacter baumannii strain 2024CK-01227 chromosome, complete genome | Acinetobacter baumannii | 401 | 401 | 100,00% | 14 | 10000 | 3941216 |
| Acinetobacter baumannii strain JRCGR-ACBMN01 chromosome | Acinetobacter baumannii | 401 | 401 | 100,00% | 14 | 10000 | 3898738 |
| Acinetobacter baumannii strain F19-02 chromosome, complete genome | Acinetobacter baumannii | 401 | 401 | 100,00% | 14 | 10000 | 3974547 |
| Acinetobacter baumannii strain 1326580 chromosome, complete genome | Acinetobacter baumannii | 401 | 401 | 100,00% | 14 | 10000 | 3870190 |
| Acinetobacter baumannii strain Rp779 chromosome, complete genome | Acinetobacter baumannii | 401 | 401 | 100,00% | 14 | 10000 | 3728679 |
| Acinetobacter baumannii strain 2022CK-00241 chromosome, complete genome | Acinetobacter baumannii | 401 | 401 | 100,00% | 14 | 10000 | 3939250 |
| Acinetobacter baumannii strain X4-705 chromosome, complete genome | Acinetobacter baumannii | 401 | 401 | 100,00% | 14 | 10000 | 3971992 |
| Acinetobacter baumannii strain XH1032 chromosome, complete genome | Acinetobacter baumannii | 401 | 724 | 100,00% | 14 | 10000 | 3929767 |
| Acinetobacter baumannii strain F14-11 chromosome, complete genome | Acinetobacter baumannii | 401 | 401 | 100,00% | 14 | 10000 | 4061790 |

| ***Yersinia enterocolitica* (YE)** | **Forward: GGTGCAGAAGCCTGGACTGA** | | | |  |  |  |
| --- | --- | --- | --- | --- | --- | --- | --- |
| **Description** | **Scientific Name** | **Max Score** | **Total Score** | **Query Cover** | **E value** | **Per. ident** | **Acc. Len** |
| Yersinia enterocolitica strain Y155 chromosome, complete genome | Yersinia enterocolitica | 401 | 401 | 100,00% | 14 | 10000 | 4605723 |
| Yersinia enterocolitica strain NW116 chromosome | Yersinia enterocolitica | 401 | 401 | 100,00% | 14 | 10000 | 4770099 |
| Yersinia enterocolitica strain 8081, complete genome | Yersinia enterocolitica | 401 | 401 | 100,00% | 14 | 10000 | 4616187 |
| Yersinia enterocolitica strain Y201 chromosome, complete genome | Yersinia enterocolitica | 401 | 401 | 100,00% | 14 | 10000 | 4839737 |
| Yersinia enterocolitica strain MP98 chromosome | Yersinia enterocolitica | 401 | 401 | 100,00% | 14 | 10000 | 4794701 |
| Yersinia enterocolitica strain WA, complete genome | Yersinia enterocolitica | 401 | 401 | 100,00% | 14 | 10000 | 4548749 |
| Yersinia enterocolitica strain NW67 chromosome | Yersinia enterocolitica | 401 | 401 | 100,00% | 14 | 10000 | 4821345 |
| Yersinia enterocolitica strain Y177 chromosome, complete genome | Yersinia enterocolitica | 401 | 401 | 100,00% | 14 | 10000 | 4605718 |
| Yersinia enterocolitica strain FORC_002, complete genome | Yersinia enterocolitica | 401 | 401 | 100,00% | 14 | 10000 | 4735535 |
| Yersinia enterocolitica W22703 biovar 2, serovar O:9, contig 7180000001361 | Yersinia enterocolitica W22703 | 401 | 401 | 100,00% | 14 | 10000 | 18886 |

| ***Yersinia enterocolitica* (YE)** | **Reverse: CACCCAACTGTGGAAGTGCAG** | | | |  |  |  |
| --- | --- | --- | --- | --- | --- | --- | --- |
| **Description** | **Scientific Name** | **Max Score** | **Total Score** | **Query Cover** | **E value** | **Per. ident** | **Acc. Len** |
| Yersinia enterocolitica strain Y154 chromosome, complete genome | Yersinia enterocolitica | 421 | 421 | 100,00% | 35 | 10000 | 4737654 |
| Yersinia enterocolitica strain Y155 chromosome, complete genome | Yersinia enterocolitica | 421 | 421 | 100,00% | 35 | 10000 | 4605723 |
| Yersinia enterocolitica strain NW1 chromosome | Yersinia enterocolitica | 421 | 421 | 100,00% | 35 | 10000 | 4737272 |
| Yersinia enterocolitica strain NW116 chromosome | Yersinia enterocolitica | 421 | 421 | 100,00% | 35 | 10000 | 4770099 |
| Yersinia enterocolitica strain C760 invasin (invA) gene, partial cds | Yersinia enterocolitica | 421 | 421 | 100,00% | 35 | 10000 | 1060 |
| Yersinia enterocolitica strain Y201 chromosome, complete genome | Yersinia enterocolitica | 421 | 421 | 100,00% | 35 | 10000 | 4839737 |
| Yersinia enterocolitica strain MP98 chromosome | Yersinia enterocolitica | 421 | 421 | 100,00% | 35 | 10000 | 4794701 |
| Yersinia enterocolitica strain Gp200 chromosome | Yersinia enterocolitica | 421 | 421 | 100,00% | 35 | 10000 | 4530554 |
| Yersinia enterocolitica strain NW67 chromosome | Yersinia enterocolitica | 421 | 421 | 100,00% | 35 | 10000 | 4821345 |
| Yersinia enterocolitica strain Y177 chromosome, complete genome | Yersinia enterocolitica | 421 | 421 | 100,00% | 35 | 10000 | 4605718 |

| ***Enterococcus faecalis* (EF)** | **Forward: TGGACAACCAACTGGCGCTAT** | | | |  |  |  |
| --- | --- | --- | --- | --- | --- | --- | --- |
| **Description** | **Scientific Name** | **Max Score** | **Total Score** | **Query Cover** | **E value** | **Per. ident** | **Acc. Len** |
| Enterococcus faecalis strain EfsC49 chromosome, complete genome | Enterococcus faecalis | 421 | 421 | 100,00% | 35 | 10000 | 2765943 |
| Enterococcus faecalis strain Fac74 chromosome, complete genome | Enterococcus faecalis | 421 | 421 | 100,00% | 35 | 10000 | 3049549 |
| Enterococcus faecalis strain J-5-A chromosome, complete genome | Enterococcus faecalis | 421 | 421 | 100,00% | 35 | 10000 | 2866940 |
| Enterococcus faecalis AS11 sodA gene for superoxide dismutase, partial cds | Enterococcus faecalis | 421 | 421 | 100,00% | 35 | 10000 | 249 |
| Enterococcus faecalis strain PF_L2_EN_IN chromosome | Enterococcus faecalis | 421 | 421 | 100,00% | 35 | 10000 | 2806553 |
| Enterococcus faecalis AS17 sodA gene for superoxide dismutase, partial cds | Enterococcus faecalis | 421 | 421 | 100,00% | 35 | 10000 | 301 |
| Enterococcus faecalis strain AT09 chromosome, complete genome | Enterococcus faecalis | 421 | 421 | 100,00% | 35 | 10000 | 2836336 |
| Enterococcus faecalis strain EfsPF13 chromosome, complete genome | Enterococcus faecalis | 421 | 421 | 100,00% | 35 | 10000 | 2870187 |
| Enterococcus faecalis strain DJH702 chromosome, complete genome | Enterococcus faecalis | 421 | 421 | 100,00% | 35 | 10000 | 2884316 |
| Enterococcus faecalis strain N775_PC2 chromosome, complete genome | Enterococcus faecalis | 421 | 421 | 100,00% | 35 | 10000 | 2865778 |

| ***Enterococcus faecalis* (EF)** | **Reverse: CCAAGCCCAACCTGAACCAA** | | | |  |  |  |
| --- | --- | --- | --- | --- | --- | --- | --- |
| **Description** | **Scientific Name** | **Max Score** | **Total Score** | **Query Cover** | **E value** | **Per. ident** | **Acc. Len** |
| Staphylococcus aureus strain RIVM_M047065 chromosome, complete genome | Staphylococcus aureus | 401 | 401 | 100,00% | 14 | 10000 | 2806671 |
| Staphylococcus aureus strain 21 chromosome | Staphylococcus aureus | 401 | 401 | 100,00% | 14 | 10000 | 2822992 |
| Staphylococcus aureus strain BSN123 chromosome, complete genome | Staphylococcus aureus | 401 | 401 | 100,00% | 14 | 10000 | 2911884 |
| Flavobacterium sp. TBRC 19031 chromosome, complete genome | Flavobacterium sp. TBRC 19031 | 401 | 401 | 100,00% | 14 | 10000 | 3023459 |
| Staphylococcus aureus strain C896 chromosome, complete genome | Staphylococcus aureus | 401 | 401 | 100,00% | 14 | 10000 | 2802834 |
| Staphylococcus aureus strain C324 chromosome, complete genome | Staphylococcus aureus | 401 | 401 | 100,00% | 14 | 10000 | 2826995 |
| Staphylococcus aureus strain C870 chromosome, complete genome | Staphylococcus aureus | 401 | 401 | 100,00% | 14 | 10000 | 2811242 |
| Staphylococcus aureus strain C364 chromosome, complete genome | Staphylococcus aureus | 401 | 401 | 100,00% | 14 | 10000 | 2700378 |
| Staphylococcus aureus strain AG21-0610 chromosome, complete genome | Staphylococcus aureus | 401 | 401 | 100,00% | 14 | 10000 | 2919215 |
| Staphylococcus aureus strain 2.1 chromosome, complete genome | Staphylococcus aureus | 401 | 401 | 100,00% | 14 | 10000 | 2722329 |
| Staphylococcus aureus strain CNRS23739 chromosome | Staphylococcus aureus | 401 | 401 | 100,00% | 14 | 10000 | 2843863 |
| Staphylococcus aureus strain SA27-SX chromosome, complete genome | Staphylococcus aureus | 401 | 401 | 100,00% | 14 | 10000 | 2808217 |
| Staphylococcus aureus strain UNC_SA54 chromosome, complete genome | Staphylococcus aureus | 401 | 401 | 100,00% | 14 | 10000 | 2755141 |
| Aliarcobacter butzleri strain BNI-3167 chromosome, complete genome | Aliarcobacter butzleri | 401 | 401 | 100,00% | 14 | 10000 | 2168578 |
| Enterococcus faecalis strain BE54 chromosome, complete genome | Enterococcus faecalis | 401 | 401 | 100,00% | 14 | 10000 | 2978606 |
| Flavobacterium psychrophilum strain 2022-516 chromosome, complete genome | Flavobacterium psychrophilum | 401 | 724 | 100,00% | 14 | 10000 | 2856107 |
| Staphylococcus aureus strain B0354 chromosome, complete genome | Staphylococcus aureus | 401 | 401 | 100,00% | 14 | 10000 | 2921834 |
| Staphylococcus aureus strain BSN77 chromosome, complete genome | Staphylococcus aureus | 401 | 401 | 100,00% | 14 | 10000 | 2918960 |
| Staphylococcus simulans strain IVB6179 chromosome, complete genome | Staphylococcus simulans | 401 | 401 | 100,00% | 14 | 10000 | 2662365 |
| Staphylococcus aureus strain OD028 chromosome, complete genome | Staphylococcus aureus | 401 | 401 | 100,00% | 14 | 10000 | 2473420 |
| Staphylococcus aureus strain C69 chromosome, complete genome | Staphylococcus aureus | 401 | 401 | 100,00% | 14 | 10000 | 2874724 |
| MAG: uncultured Sunxiuqinia sp. isolate ca0ff389-0d66-4f98-8792-5b2e3713dedf genome assembly, chromosome: 2 | uncultured Sunxiuqinia sp. | 401 | 401 | 100,00% | 14 | 10000 | 1102336 |
| Arcobacter sp. KX21116 chromosome, complete genome | Arcobacter sp. KX21116 | 401 | 401 | 100,00% | 14 | 10000 | 3158278 |
| Staphylococcus aureus strain C325 chromosome, complete genome | Staphylococcus aureus | 401 | 401 | 100,00% | 14 | 10000 | 2823073 |
| Staphylococcus aureus strain BSN102 chromosome, complete genome | Staphylococcus aureus | 401 | 401 | 100,00% | 14 | 10000 | 2840121 |
| Staphylococcus aureus strain 15 chromosome, complete genome | Staphylococcus aureus | 401 | 401 | 100,00% | 14 | 10000 | 2799886 |
| Staphylococcus aureus strain C137 chromosome, complete genome | Staphylococcus aureus | 401 | 401 | 100,00% | 14 | 10000 | 2776891 |
| Staphylococcus aureus strain TUM22721 chromosome, complete genome | Staphylococcus aureus | 401 | 401 | 100,00% | 14 | 10000 | 2825857 |
| Staphylococcus aureus strain Viktor chromosome, complete genome | Staphylococcus aureus | 401 | 401 | 100,00% | 14 | 10000 | 2878679 |
| Staphylococcus aureus strain Sau86 chromosome, complete genome | Staphylococcus aureus | 401 | 401 | 100,00% | 14 | 10000 | 2895676 |
| Staphylococcus aureus strain NRS384 chromosome, complete genome | Staphylococcus aureus | 401 | 401 | 100,00% | 14 | 10000 | 2879034 |
| Staphylococcus aureus strain BE-MSSA6 chromosome, complete genome | Staphylococcus aureus | 401 | 401 | 100,00% | 14 | 10000 | 2692770 |
| Streptococcus agalactiae strain HU36/21 chromosome, complete genome | Streptococcus agalactiae | 401 | 401 | 100,00% | 14 | 10000 | 2130023 |
| Globicatella sanguinis strain UMB0514 chromosome, complete genome | Globicatella sanguinis | 401 | 401 | 100,00% | 14 | 10000 | 2622382 |
| Staphylococcus aureus strain MSSA-JAR chromosome, complete genome | Staphylococcus aureus | 401 | 401 | 100,00% | 14 | 10000 | 2746087 |
| Staphylococcus aureus strain OD001 chromosome, complete genome | Staphylococcus aureus | 401 | 401 | 100,00% | 14 | 10000 | 2479797 |
| Staphylococcus aureus strain Sau34 chromosome, complete genome | Staphylococcus aureus | 401 | 401 | 100,00% | 14 | 10000 | 2868342 |
| Staphylococcus aureus strain B0356 chromosome, complete genome | Staphylococcus aureus | 401 | 401 | 100,00% | 14 | 10000 | 2776580 |
| Staphylococcus aureus strain BSN124 chromosome, complete genome | Staphylococcus aureus | 401 | 401 | 100,00% | 14 | 10000 | 2889032 |
| Staphylococcus aureus strain 808 chromosome | Staphylococcus aureus | 401 | 401 | 100,00% | 14 | 10000 | 2861752 |
| Staphylococcus aureus 59458 DNA, complete genome | Staphylococcus aureus | 401 | 401 | 100,00% | 14 | 10000 | 2849402 |
| Staphylococcus aureus strain Dog043 chromosome, complete genome | Staphylococcus aureus | 401 | 401 | 100,00% | 14 | 10000 | 2783847 |
| Staphylococcus aureus strain ER21336.3 chromosome, complete genome | Staphylococcus aureus | 401 | 401 | 100,00% | 14 | 10000 | 2967435 |
| Staphylococcus aureus strain 20 chromosome | Staphylococcus aureus | 401 | 401 | 100,00% | 14 | 10000 | 2822821 |
| Aliarcobacter butzleri strain BNI-4356 chromosome, complete genome | Aliarcobacter butzleri | 401 | 401 | 100,00% | 14 | 10000 | 2184148 |
| Staphylococcus aureus strain BSN193 chromosome, complete genome | Staphylococcus aureus | 401 | 401 | 100,00% | 14 | 10000 | 2877338 |
| Staphylococcus aureus strain BSN12 chromosome, complete genome | Staphylococcus aureus | 401 | 401 | 100,00% | 14 | 10000 | 2848141 |
| Staphylococcus aureus strain C292 chromosome, complete genome | Staphylococcus aureus | 401 | 401 | 100,00% | 14 | 10000 | 2737087 |
| Staphylococcus aureus strain Dog139 chromosome, complete genome | Staphylococcus aureus | 401 | 401 | 100,00% | 14 | 10000 | 2783708 |
| Enterococcus faecalis strain EfsC20 chromosome, complete genome | Enterococcus faecalis | 401 | 401 | 100,00% | 14 | 10000 | 2904406 |
| Lactococcus petauri strain R21-69 HC chromosome, complete genome | Lactococcus petauri | 401 | 401 | 100,00% | 14 | 10000 | 2112152 |
| Staphylococcus aureus strain sa230711_barcode73 chromosome, complete genome | Staphylococcus aureus | 401 | 401 | 100,00% | 14 | 10000 | 2727015 |
| Staphylococcus aureus strain 0073_III_ST239 chromosome, complete genome | Staphylococcus aureus | 401 | 401 | 100,00% | 14 | 10000 | 3070202 |
| Staphylococcus aureus strain B0421 chromosome, complete genome | Staphylococcus aureus | 401 | 401 | 100,00% | 14 | 10000 | 2727740 |
| Staphylococcus aureus strain C62 chromosome, complete genome | Staphylococcus aureus | 401 | 401 | 100,00% | 14 | 10000 | 2884808 |
| Staphylococcus aureus strain JP18270 chromosome, complete genome | Staphylococcus aureus | 401 | 401 | 100,00% | 14 | 10000 | 2732260 |
| Staphylococcus simulans strain DRD-33 chromosome | Staphylococcus simulans | 401 | 401 | 100,00% | 14 | 10000 | 2362102 |
| Staphylococcus aureus strain BSN203 chromosome | Staphylococcus aureus | 401 | 401 | 100,00% | 14 | 10000 | 2769416 |
| Enterococcus faecalis strain Z81-6 chromosome | Enterococcus faecalis | 401 | 401 | 100,00% | 14 | 10000 | 2875735 |
| Staphylococcus aureus strain BSN67 chromosome, complete genome | Staphylococcus aureus | 401 | 401 | 100,00% | 14 | 10000 | 2789706 |
| Staphylococcus aureus strain BSN136 chromosome | Staphylococcus aureus | 401 | 401 | 100,00% | 14 | 10000 | 2858048 |
| Staphylococcus aureus strain BSN46-2 chromosome, complete genome | Staphylococcus aureus | 401 | 401 | 100,00% | 14 | 10000 | 2920103 |
| Aliarcobacter butzleri strain P1200 chromosome, complete genome | Aliarcobacter butzleri | 401 | 401 | 100,00% | 14 | 10000 | 2143012 |
| Staphylococcus aureus strain BSN15 chromosome, complete genome | Staphylococcus aureus | 401 | 401 | 100,00% | 14 | 10000 | 2902857 |
| Flavobacterium psychrophilum strain 2022-465 chromosome, complete genome | Flavobacterium psychrophilum | 401 | 724 | 100,00% | 14 | 10000 | 2856105 |
| Enterococcus faecalis strain HL1 chromosome, complete genome | Enterococcus faecalis | 401 | 401 | 100,00% | 14 | 10000 | 2695805 |
| Staphylococcus aureus strain SA1807 chromosome, complete genome | Staphylococcus aureus | 401 | 401 | 100,00% | 14 | 10000 | 2849387 |
| Staphylococcus aureus strain C280 chromosome, complete genome | Staphylococcus aureus | 401 | 401 | 100,00% | 14 | 10000 | 2805792 |
| Staphylococcus argenteus strain RIVM_M046968 chromosome, complete genome | Staphylococcus argenteus | 401 | 401 | 100,00% | 14 | 10000 | 2762566 |
| Staphylococcus aureus strain BSN196 chromosome | Staphylococcus aureus | 401 | 401 | 100,00% | 14 | 10000 | 2922534 |
| Staphylococcus aureus strain SA2166 chromosome, complete genome | Staphylococcus aureus | 401 | 401 | 100,00% | 14 | 10000 | 2689085 |
| Staphylococcus aureus strain Sau69 chromosome, complete genome | Staphylococcus aureus | 401 | 401 | 100,00% | 14 | 10000 | 2929725 |
| Staphylococcus aureus strain BSN105 chromosome | Staphylococcus aureus | 401 | 401 | 100,00% | 14 | 10000 | 2772686 |
| Staphylococcus aureus strain C318 chromosome, complete genome | Staphylococcus aureus | 401 | 401 | 100,00% | 14 | 10000 | 2946430 |
| Staphylococcus aureus strain BSN69 chromosome, complete genome | Staphylococcus aureus | 401 | 401 | 100,00% | 14 | 10000 | 2872019 |
| Staphylococcus aureus strain BSN205 chromosome, complete genome | Staphylococcus aureus | 401 | 401 | 100,00% | 14 | 10000 | 2887530 |
| Staphylococcus aureus strain BSN47-2 chromosome, complete genome | Staphylococcus aureus | 401 | 401 | 100,00% | 14 | 10000 | 2918888 |
| Staphylococcus aureus strain 137G chromosome, complete genome | Staphylococcus aureus | 401 | 401 | 100,00% | 14 | 10000 | 2791835 |
| Staphylococcus aureus strain WA121-2021_15363 chromosome, complete genome | Staphylococcus aureus | 401 | 401 | 100,00% | 14 | 10000 | 2840551 |
| Staphylococcus aureus strain BSN122 chromosome, complete genome | Staphylococcus aureus | 401 | 401 | 100,00% | 14 | 10000 | 2760105 |
| Staphylococcus aureus strain CUBIST-17 chromosome, complete genome | Staphylococcus aureus | 401 | 401 | 100,00% | 14 | 10000 | 2877074 |
| Staphylococcus aureus strain C201 chromosome, complete genome | Staphylococcus aureus | 401 | 401 | 100,00% | 14 | 10000 | 2867965 |
| Staphylococcus aureus CN09 DNA, complete genome | Staphylococcus aureus | 401 | 401 | 100,00% | 14 | 10000 | 2860583 |
| Flavobacterium psychrophilum strain 160401-1/5N-R9 chromosome | Flavobacterium psychrophilum | 401 | 724 | 100,00% | 14 | 10000 | 2827643 |
| Staphylococcus aureus strain BSN202 chromosome, complete genome | Staphylococcus aureus | 401 | 401 | 100,00% | 14 | 10000 | 2800495 |
| Staphylococcus aureus strain Dog103 chromosome, complete genome | Staphylococcus aureus | 401 | 401 | 100,00% | 14 | 10000 | 2783696 |
| Staphylococcus aureus strain NCCP11854 chromosome, complete genome | Staphylococcus aureus | 401 | 401 | 100,00% | 14 | 10000 | 2750982 |
| Staphylococcus aureus strain CNRS22769 chromosome | Staphylococcus aureus | 401 | 401 | 100,00% | 14 | 10000 | 2844863 |
| Staphylococcus aureus strain BSN197 chromosome, complete genome | Staphylococcus aureus | 401 | 401 | 100,00% | 14 | 10000 | 2875597 |
| Staphylococcus aureus strain IVB6154 chromosome, complete genome | Staphylococcus aureus | 401 | 401 | 100,00% | 14 | 10000 | 2865130 |
| Staphylococcus aureus strain TUM20817 chromosome, complete genome | Staphylococcus aureus | 401 | 401 | 100,00% | 14 | 10000 | 2824635 |
| Staphylococcus aureus strain Sau147 chromosome, complete genome | Staphylococcus aureus | 401 | 401 | 100,00% | 14 | 10000 | 3000039 |
| Staphylococcus aureus strain CBTW2018367 chromosome, complete genome | Staphylococcus aureus | 401 | 401 | 100,00% | 14 | 10000 | 2833324 |
| Enterococcus faecalis strain DJH702_8 chromosome, complete genome | Enterococcus faecalis | 401 | 401 | 100,00% | 14 | 10000 | 2884962 |
| Staphylococcus aureus strain Dog105 chromosome, complete genome | Staphylococcus aureus | 401 | 401 | 100,00% | 14 | 10000 | 2783699 |
| Staphylococcus aureus strain SCTW2019099 chromosome, complete genome | Staphylococcus aureus | 401 | 401 | 100,00% | 14 | 10000 | 2755432 |
| Staphylococcus aureus strain ATCC 29213 chromosome, complete genome | Staphylococcus aureus | 401 | 401 | 100,00% | 14 | 10000 | 2762291 |
| Enterococcus faecalis strain BE65 chromosome, complete genome | Enterococcus faecalis | 401 | 401 | 100,00% | 14 | 10000 | 2963973 |
| Staphylococcus aureus strain TUM22705 chromosome, complete genome | Staphylococcus aureus | 401 | 401 | 100,00% | 14 | 10000 | 2784767 |
| Staphylococcus aureus strain TUM22178 chromosome, complete genome | Staphylococcus aureus | 401 | 401 | 100,00% | 14 | 10000 | 2818904 |

| ***Pseudomonas viridiflava* (PV)** | **Forward: CGTAGGCGAGAGCGATACCA** | | | |  |  |  |
| --- | --- | --- | --- | --- | --- | --- | --- |
| **Description** | **Scientific Name** | **Max Score** | **Total Score** | **Query Cover** | **E value** | **Per. ident** | **Acc. Len** |
| Pseudomonas viridiflava gyrB gene for DNA gyrase subunit B, partial cds, strain: MAFF 302658 | Pseudomonas viridiflava | 401 | 401 | 100,00% | 14 | 10000 | 529 |
| Pseudomonas viridiflava gyrB gene for DNA gyrase subunit B, partial cds, strain: M1-1F | Pseudomonas viridiflava | 401 | 401 | 100,00% | 14 | 10000 | 529 |
| Pseudomonas viridiflava strain MHPV2203 DNA gyrase subunit B (gyrB) gene, partial cds | Pseudomonas viridiflava | 401 | 401 | 100,00% | 14 | 10000 | 800 |
| Pseudomonas viridiflava strain LPPA 1598 DNA gyrase subunit B (gyrB) gene, partial cds | Pseudomonas viridiflava | 401 | 401 | 100,00% | 14 | 10000 | 610 |
| Pseudomonas viridiflava strain LPPA362 DNA gyrase subunit B (gyrB) gene, partial cds | Pseudomonas viridiflava | 401 | 401 | 100,00% | 14 | 10000 | 741 |
| Pseudomonas viridiflava strain G-2 DNA gyrase subunit B (gyrB) gene, complete cds | Pseudomonas viridiflava | 401 | 401 | 100,00% | 14 | 10000 | 2418 |
| Pseudomonas viridiflava strain BC2508 DNA gyrase subunit B (gyrB) gene, partial cds | Pseudomonas viridiflava | 401 | 401 | 100,00% | 14 | 10000 | 638 |
| Pseudomonas viridiflava strain LPPA 1604 DNA gyrase subunit B (gyrB) gene, partial cds | Pseudomonas viridiflava | 401 | 401 | 100,00% | 14 | 10000 | 610 |
| Pseudomonas viridiflava strain LPPA74 DNA gyrase subunit B (gyrB) gene, partial cds | Pseudomonas viridiflava | 401 | 401 | 100,00% | 14 | 10000 | 741 |
| Pseudomonas viridiflava strain CFBP 1590 genome assembly, chromosome: I | Pseudomonas viridiflava | 401 | 401 | 100,00% | 14 | 10000 | 6093513 |

| ***Pseudomonas viridiflava* (PV)** | **Reverse: ATGATGCCGACGCCAGAGTT** | | | |  |  |  |
| --- | --- | --- | --- | --- | --- | --- | --- |
| **Description** | **Scientific Name** | **Max Score** | **Total Score** | **Query Cover** | **E value** | **Per. ident** | **Acc. Len** |
| Pseudomonas viridiflava gyrB gene for DNA gyrase subunit B, partial cds, strain: MAFF 302658 | Pseudomonas viridiflava | 401 | 401 | 100,00% | 14 | 10000 | 529 |
| Pseudomonas viridiflava strain K005_3_1b DNA gyrase subunit B (gyrB) gene, partial cds | Pseudomonas viridiflava | 401 | 401 | 100,00% | 14 | 10000 | 636 |
| Pseudomonas viridiflava strain SV1779 chromosome | Pseudomonas viridiflava | 401 | 401 | 100,00% | 14 | 10000 | 6106689 |
| Pseudomonas viridiflava strain LPPA 1446 DNA gyrase subunit B (gyrB) gene, partial cds | Pseudomonas viridiflava | 401 | 401 | 100,00% | 14 | 10000 | 610 |
| Pseudomonas viridiflava strain PV271 gyrase B (gyrB) gene, partial cds | Pseudomonas viridiflava | 401 | 401 | 100,00% | 14 | 10000 | 835 |
| Pseudomonas viridiflava strain CFBP 1590 genome assembly, chromosome: I | Pseudomonas viridiflava | 401 | 401 | 100,00% | 14 | 10000 | 6093513 |
| Pseudomonas viridiflava strain LPPA 846 DNA gyrase subunit B (gyrB) gene, partial cds | Pseudomonas viridiflava | 401 | 401 | 100,00% | 14 | 10000 | 610 |
| Pseudomonas viridiflava strain LPPA 941 DNA gyrase subunit B (gyrB) gene, partial cds | Pseudomonas viridiflava | 401 | 401 | 100,00% | 14 | 10000 | 610 |
| Pseudomonas viridiflava isolate KY5.1a DNA gyrase subunit B (gyrB) gene, partial cds | Pseudomonas viridiflava | 401 | 401 | 100,00% | 14 | 10000 | 741 |
| Pseudomonas viridiflava strain K005_3_1c DNA gyrase subunit B (gyrB) gene, partial cds | Pseudomonas viridiflava | 401 | 401 | 100,00% | 14 | 10000 | 636 |

| ***Xanthomonas vesicatoria* (XV)** | **Forward: GTGCTGTCTCTGCGGGAATG** | | | |  |  |  |
| --- | --- | --- | --- | --- | --- | --- | --- |
| **Description** | **Scientific Name** | **Max Score** | **Total Score** | **Query Cover** | **E value** | **Per. ident** | **Acc. Len** |
| Xanthomonas vesicatoria ATCC 35937 strain LMG911 chromosome, complete genome | Xanthomonas vesicatoria ATCC 35937 | 401 | 401 | 100,00% | 14 | 10000 | 5453503 |
| Xanthomonas vesicatoria strain WHRI 8302 chromosome, complete genome | Xanthomonas vesicatoria | 401 | 401 | 100,00% | 14 | 10000 | 5110163 |
| Spinus spinus genome assembly, chromosome: 11 | Spinus spinus | 382 | 704 | 95,00% | 55 | 10000 | 5007668 |
| Okeanomitos corallinicola TIOX110 chromosome, complete genome | Okeanomitos corallinicola TIOX110 | 382 | 382 | 95,00% | 55 | 10000 | 1797862 |
| Spinus spinus genome assembly, chromosome: 11 | Spinus spinus | 382 | 704 | 95,00% | 55 | 10000 | 6564943 |
| Geomonas nitrogeniifigens strain RF4 chromosome, complete genome | Geomonas nitrogeniifigens | 362 | 362 | 90,00% | 22 | 10000 | 6361125 |
| Microcoleus vaginatus HSN003, whole genome shotgun sequence | Microcoleus vaginatus HSN003 | 362 | 362 | 90,00% | 22 | 10000 | 6,7E+07 |
| Brevibacillus humidisoli strain MMS20-4M-10-Y chromosome, complete genome | Brevibacillus humidisoli | 362 | 362 | 90,00% | 22 | 10000 | 6943 |
| Microcoleus vaginatus PCC 9802 chromosome, complete genome | Microcoleus vaginatus PCC 9802 | 362 | 362 | 90,00% | 22 | 10000 | 2043 |
| Geomonas nitrogeniifigens strain RG29 chromosome, complete genome | Geomonas nitrogeniifigens | 362 | 362 | 90,00% | 22 | 10000 | 6968785 |

| ***Xanthomonas vesicatoria* (XV)** | **Reverse: GGCGTAGCCAATGGTCCAAC** | | | |  |  |  |
| --- | --- | --- | --- | --- | --- | --- | --- |
| **Description** | **Scientific Name** | **Max Score** | **Total Score** | **Query Cover** | **E value** | **Per. ident** | **Acc. Len** |
| Diaphorobacter sp. HDW4B chromosome, complete genome | Diaphorobacter sp. HDW4B | 401 | 401 | 100,00% | 14 | 10000 | 5453503 |
| Xanthomonas vesicatoria ATCC 35937 strain LMG911 chromosome, complete genome | Xanthomonas vesicatoria ATCC 35937 | 401 | 401 | 100,00% | 14 | 10000 | 5110163 |
| Xanthomonas vesicatoria strain WHRI 8302 chromosome, complete genome | Xanthomonas vesicatoria | 401 | 401 | 100,00% | 14 | 10000 | 5007668 |
| Scardovia inopinata JCM 12537 DNA, complete genome | Scardovia inopinata JCM 12537 | 362 | 362 | 90,00% | 22 | 10000 | 1797862 |
| Larkinella insperata strain LMG22510 chromosome, complete genome | Larkinella insperata | 362 | 362 | 90,00% | 22 | 10000 | 6564943 |
| Spartinivicinus ruber strain S2-4-1H chromosome, complete genome | Spartinivicinus ruber | 362 | 362 | 90,00% | 22 | 10000 | 6361125 |
| Bradybaena similaris mixture of YIPCMo001421 and YIPCMo001540 DNA, chromosome 19, sequence | Bradybaena similaris | 362 | 362 | 90,00% | 22 | 10000 | 6,7E+07 |
| PREDICTED: Drosophila innubila uncharacterized LOC117792415 (LOC117792415), mRNA | Drosophila innubila | 342 | 342 | 85,00% | 86 | 10000 | 6943 |
| Fusarium keratoplasticum Amine oxidase (NCS57_00904000), partial mRNA | Fusarium keratoplasticum | 342 | 342 | 85,00% | 86 | 10000 | 2043 |
| Pseudomonas aeruginosa strain Li010 chromosome, complete genome | Pseudomonas aeruginosa | 342 | 342 | 85,00% | 86 | 10000 | 6968785 |

| ***Rhodococcus fascians* (RF)** | **Forward: TCGTTGACTCTCGCTCTGCT** | | | |  |  |  |
| --- | --- | --- | --- | --- | --- | --- | --- |
| **Description** | **Scientific Name** | **Max Score** | **Total Score** | **Query Cover** | **E value** | **Per. ident** | **Acc. Len** |
| Rhodococcoides fascians strain YWS3-1 chromosome, complete genome | Rhodococcoides fascians | 401 | 401 | 100,00% | 14 | 10000 | 5364829 |
| Rhodococcus fascians D188, complete genome | Rhodococcoides fascians D188 | 401 | 401 | 100,00% | 14 | 10000 | 5139988 |
| PREDICTED: Amphimedon queenslandica uncharacterized LOC109591407 (LOC109591407), partial mRNA | Amphimedon queenslandica | 401 | 401 | 100,00% | 14 | 10000 | 670 |
| Rhodococcus sp. PBTS2, complete genome | Rhodococcoides fascians | 401 | 401 | 100,00% | 14 | 10000 | 5179353 |
| PREDICTED: Amphimedon queenslandica flocculation protein FLO11-like (LOC109585168), mRNA | Amphimedon queenslandica | 401 | 401 | 100,00% | 14 | 10000 | 4331 |
| PREDICTED: Armigeres subalbatus coiled-coil domain-containing protein 22 homolog (LOC134227120), mRNA | Armigeres subalbatus | 382 | 382 | 95,00% | 55 | 10000 | 2158 |
| Fusarium tjaetaba polyketide cyclase (FTJAE_2011), partial mRNA | Fusarium tjaetaba | 362 | 362 | 90,00% | 22 | 10000 | 807 |
| Apaeleticus inimicus genome assembly, chromosome: 9 | Unknown | 362 | 362 | 90,00% | 22 | 10000 | 1,9E+07 |
| PREDICTED: Linepithema humile discs large 1 (dlg1), transcript variant X3, mRNA | Linepithema humile | 362 | 362 | 90,00% | 22 | 10000 | 8762 |
| Fusarium verticillioides 7600 hypothetical protein (FVEG_11662), partial mRNA | Fusarium verticillioides 7600 | 362 | 362 | 90,00% | 22 | 10000 | 798 |

| ***Rhodococcus fascians* (RF)** | **Reverse: CACCAATGCCGTCGGGTATC** | | | |  |  |  |
| --- | --- | --- | --- | --- | --- | --- | --- |
| **Description** | **Scientific Name** | **Max Score** | **Total Score** | **Query Cover** | **E value** | **Per. ident** | **Acc. Len** |
| Rhodococcus fascians D188, complete genome | Rhodococcoides fascians D188 | 401 | 401 | 100,00% | 14 | 10000 | 5139988 |
| Pseudomonas simiae strain PICF7, complete genome | Pseudomonas simiae | 382 | 382 | 95,00% | 55 | 10000 | 6136735 |
| Pseudomonas simiae strain H4-B59 chromosome, complete genome | Pseudomonas simiae | 382 | 382 | 95,00% | 55 | 10000 | 6079809 |
| Pseudomonas simiae strain FP55 chromosome, complete genome | Pseudomonas simiae | 382 | 382 | 95,00% | 55 | 10000 | 6098551 |
| Pseudomonas simiae strain PCL1751, complete genome | Pseudomonas simiae | 382 | 382 | 95,00% | 55 | 10000 | 6143950 |
| Pseudomonas simiae strain WCS417 genome | Pseudomonas simiae | 382 | 382 | 95,00% | 55 | 10000 | 6169071 |
| Pseudomonas simiae strain K-Hf-L9 chromosome, complete genome | Pseudomonas simiae | 382 | 382 | 95,00% | 55 | 10000 | 6199521 |
| Pseudomonas simiae strain POE78A chromosome, complete genome | Pseudomonas simiae | 382 | 382 | 95,00% | 55 | 10000 | 6225905 |
| Pseudomonas simiae strain FP2084 chromosome, complete genome | Pseudomonas simiae | 382 | 382 | 95,00% | 55 | 10000 | 6404655 |
| Pseudomonas simiae strain FP1885 chromosome, complete genome | Pseudomonas simiae | 382 | 382 | 95,00% | 55 | 10000 | 6297891 |

| ***Pseudomonas syringae* (PS)** | **Forward: AGTTTCAGCACGCCAGCAAA** | | | |  |  |  |
| --- | --- | --- | --- | --- | --- | --- | --- |
| **Description** | **Scientific Name** | **Max Score** | **Total Score** | **Query Cover** | **E value** | **Per. ident** | **Acc. Len** |
| Pseudomonas avellanae strain NCPPB 4222 chromosome, complete genome | Pseudomonas avellanae | 401 | 401 | 100,00% | 14 | 10000 | 6062886 |
| Pseudomonas syringae pv. coryli hrpL gene for sigma factor, strain DPP51 | Pseudomonas syringae pv. coryli | 401 | 401 | 100,00% | 14 | 10000 | 555 |
| Pseudomonas syringae strain MUP20 chromosome, complete genome | Pseudomonas syringae | 401 | 724 | 100,00% | 14 | 10000 | 6045198 |
| Pseudomonas syringae strain RM29.1a HrpL (hrpL) gene, complete cds | Pseudomonas syringae | 401 | 401 | 100,00% | 14 | 10000 | 555 |
| Pseudomonas syringae strain CAS02 chromosome, complete genome | Pseudomonas syringae | 401 | 401 | 100,00% | 14 | 10000 | 6029081 |
| Pseudomonas syringae pv. morsprunorum hrpL gene for sigma factor, complete cds | Pseudomonas syringae | 401 | 401 | 100,00% | 14 | 10000 | 555 |
| Pseudomonas syringae pv. tomato strain GM 113 chromosome, complete genome | Pseudomonas syringae pv. tomato | 401 | 401 | 100,00% | 14 | 10000 | 6146598 |
| Pseudomonas syringae strain PNA29.1a HrpL (hrpL) gene, complete cds | Pseudomonas syringae | 401 | 401 | 100,00% | 14 | 10000 | 555 |
| Pseudomonas coronafaciens strain X-1 chromosome, complete genome | Pseudomonas coronafaciens | 401 | 401 | 100,00% | 14 | 10000 | 5661408 |
| Pseudomonas syringae pv. pisi HrpJ (hrpJ) gene, partial cds, putative sigma factor HrpL (hrpL) gene, complete cds, and HrpK (hrpK) gene, partial cds | Pseudomonas syringae pv. pisi | 401 | 401 | 100,00% | 14 | 10000 | 897 |

| ***Pseudomonas syringae* (PS)** | **Reverse: CGTTCGACTCCAGGTCCGTA** | | | |  |  |  |
| --- | --- | --- | --- | --- | --- | --- | --- |
| **Description** | **Scientific Name** | **Max Score** | **Total Score** | **Query Cover** | **E value** | **Per. ident** | **Acc. Len** |
| Pseudomonas syringae pv. coryli hrpL gene for sigma factor, strain DPP51 | Pseudomonas syringae pv. coryli | 401 | 401 | 100,00% | 14 | 10000 | 555 |
| Pseudomonas syringae strain MUP20 chromosome, complete genome | Pseudomonas syringae | 401 | 401 | 100,00% | 14 | 10000 | 6045198 |
| Pseudomonas syringae pv. pisi putative sigma factor HrpL (hrpL) gene, partial cds | Pseudomonas syringae pv. pisi | 401 | 401 | 100,00% | 14 | 10000 | 250 |
| Pseudomonas syringae pv. pisi HrpJ (hrpJ) gene, partial cds, putative sigma factor HrpL (hrpL) gene, complete cds, and HrpK (hrpK) gene, partial cds | Pseudomonas syringae pv. pisi | 401 | 401 | 100,00% | 14 | 10000 | 897 |
| Pseudomonas syringae pv. pisi hrpL gene for sigma factor, complete cds | Pseudomonas syringae pv. pisi | 401 | 401 | 100,00% | 14 | 10000 | 555 |
| Pseudomonas syringae strain KF529 chromosome, complete genome | Pseudomonas syringae | 401 | 401 | 100,00% | 14 | 10000 | 6077291 |
| Pseudomonas syringae pv. pisi BO10.2 ECF sigma 70 factor (hrpL) gene, partial cds | Pseudomonas syringae pv. pisi | 401 | 401 | 100,00% | 14 | 10000 | 250 |
| Pseudomonas syringae pv. aptata hrpL gene for sigma factor, complete cds | Pseudomonas syringae | 401 | 401 | 100,00% | 14 | 10000 | 555 |
| Pseudomonas syringae pv. pisi putative sigma factor HrpL (hrpL) gene, partial cds | Pseudomonas syringae pv. pisi | 401 | 401 | 100,00% | 14 | 10000 | 250 |
| Pseudomonas syringae pv. atrofaciens strain ARGTr 9-1 chromosome, complete genome | Pseudomonas syringae pv. atrofaciens | 401 | 401 | 100,00% | 14 | 10000 | 5996715 |
